# Reconstructing the earliest known composite-tiled roofs from the Chinese Loess Plateau

**DOI:** 10.1038/s41598-023-35299-x

**Published:** 2023-05-19

**Authors:** Yijing Xu, Jing Zhou, Jianlong Zhao, Guoke Chen, Wen Li, Mingzhi Ma, Francesca Monteith, Shengyu Liu, Minghao Peng, Andrew Bevan, Hai Zhang

**Affiliations:** 1 School of Archaeology and Museology, Peking University, 5 Summer Palace Road, Beijing, 100871 China; 2 Gansu Provincial Institute of Cultural Heritage and Archaeology, 165 Heping Road, Lanzhou, 730000 China; 3 Shaanxi Academy of Archaeology, 31 Leyou Road, Xi’an, 710054 China; 4 School of Cultural Heritage, Northwest University, 1 Xuefu Street, Xi’an, 710127 China; 5 Institute of Archaeology, University College London, 31–34 Gordon Square, London, WC1H 0PY UK

**Keywords:** Evolution, Anthropology, Archaeology, Cultural evolution

## Abstract

The origins of composite tiles, one of the oldest forms of roofing, are still unclear. This study is based on a set of over 5000 clay tile fragments excavated from a single context in the Qiaocun site on the Chinese Loess Plateau, dated to ~ 2400–2200 BCE (Early Longshan Period). By combining morphological measurement statistics, 3D modeling, computer-based simulations, and reference to historical and archaeological records, we reconstruct the earliest known composite-tile roofing techniques and demonstrate that tile production was under a low-level standardization, with manual control forming a key agent during the roofing process. The quantitative study of the composite roof tiles from Qiaocun was then placed in its archaeological context and compared with other sites on the Loess Plateau. It was found that tile-roofed buildings were, by necessity, community projects. Such structures served as nodes in larger social communication networks; additionally, their appearance was linked to intensified social complexity in public affairs during the Longshan Period. The invention of clay tiles was associated with the inception of thick rammed-earth walls which had sufficient strength to serve as load-bearing structures for heavy tiled roofs. The roof tiles excavated from Qiaocun site indicate that the Loess Plateau was a key center for the origin and spread of composite tiles and related roofing and construction methods, suggesting a Longshan–Western Zhou tradition of roofing techniques in East Asia.

## Introduction

Roof tiles constitute a key human architectural innovation. Today, tiles made of ceramic, stone, concrete, or other materials are commonly used worldwide^1^. The invention and adoption of tiled roofs are significant because they greatly improved the long-term durability, waterproofing, wind resistance, and maintenance costs of roofing^2^. This had far-reaching coevolutionary effects on other changes in the materials, shape, and structure of human dwellings and other buildings (e.g., the wide use of bricks and load-bearing structures^3^). In engaging with the origins of roof tiles and their associated construction techniques, it is possible to gain key insights into architectural and social histories.

**Figure 1:**
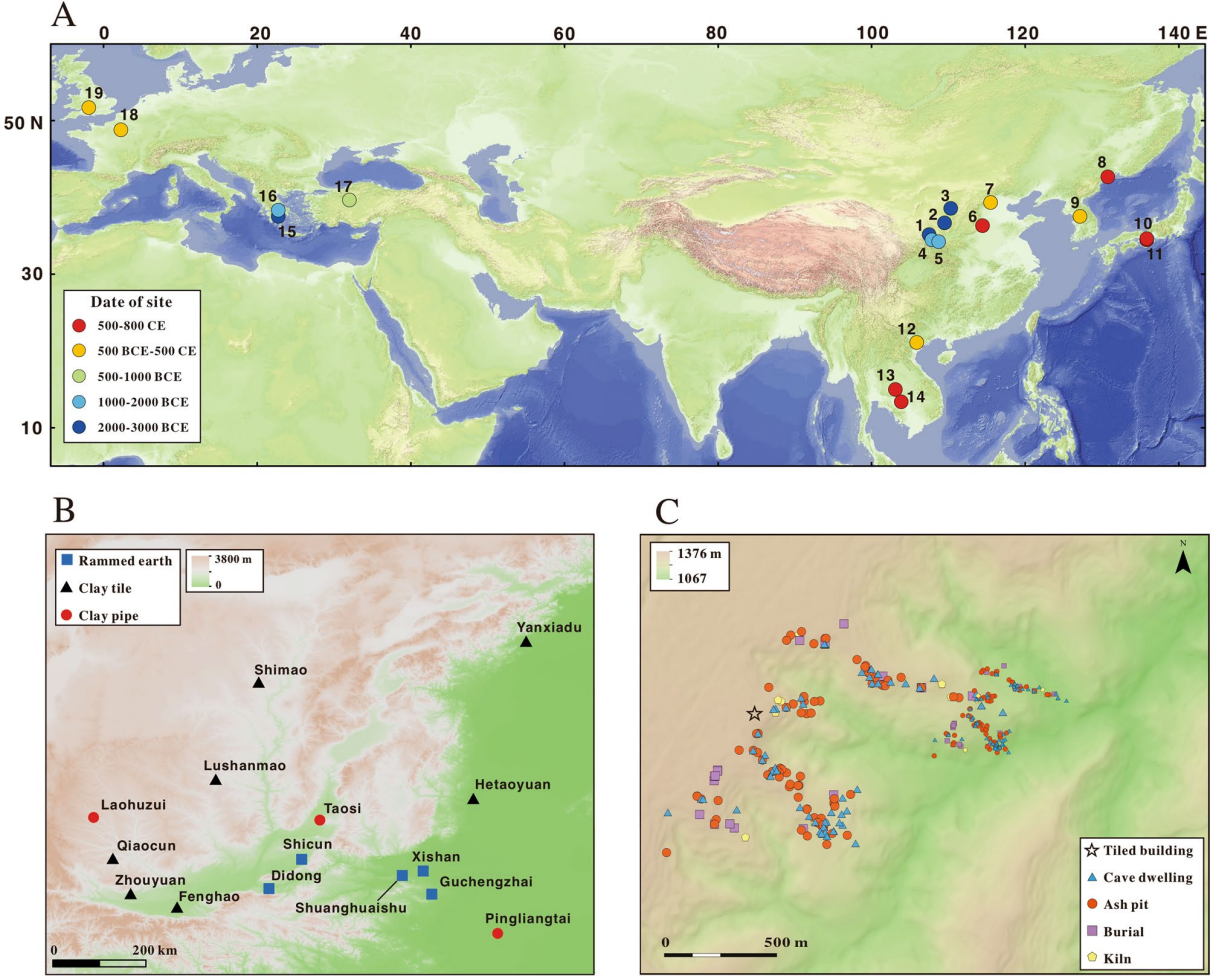
Maps of the sites with early tiles and other corresponding archaeological findings. (**A**) map of sites with early tile-roofed houses (SI Appendix, Table S4); (**B**) region of the Chinese Loess Plateau, showing sites with clay tiles, pipes and rammed earth; (**C**) map of Qiaocun site with tiled houses.

However, the origin of tiles, especially composite tiles, which consist of cover and pan tiles, to create an “over-and-under” structure have yet to be satisfactorily ascertained. The details of the earliest tiled-roofing techniques and the associated changes in architecture are still unclear. Similarly, the social background which led to the transition to tiled roofs requires investigation. Finally, did composite tile technology arise in multiple locations or did it spread from a single locale or region? Research to date has been restricted by the usually fragmentary nature of roofs and therefore roof tiles within the archaeological record. In addition, the research to date has failed to examine the social significance of the composite-tile system. On the basis of limited evidence available, previous scholars assumed that the earliest tiles were ceramic (most commonly a fired clay, referred in literature as “terracotta”) and were first used in the late third millennium BCE. In western Eurasia, such tiles are usually rectilinear with no interlocking features. They were used for the roofs of so-called “corridor houses” in settlements on the Greek mainland (~ 2650–2200 BCE)^4–6^. In eastern Eurasia, clay tiles from a similar period (Longshan Period, ~ 2400–1800 BCE) have been found in six sites at least on the Loess Plateau in China (SI Appendix, S5). Such tiles appear to have been used exclusively for larger building complexes^7,8^. It is of note that the interlocking components found in the composite-tile system in eastern Eurasia do not occur in western Eurasian contexts for another thousand years^9^ (Fig. 1A, SI Appendix, Table S4).

These archaeological discoveries suggest that the earliest roof tiles in both eastern and western Eurasia may belong to different but roughly contemporaneous technical traditions. In both cases, unusual buildings were fitted with such roofs in the context of evolving, complex local societies. In China, for example, buildings with tiled roofs are located in the center of large settlements that also retain evidence of various craft production activities and of long-distance trade (e.g., the site of Shimao which contains stone constructions and in which exotic jade objects and bone tools have been recovered, in addition to bronze production^10^). Composite-tiled roofs would appear to be an important indicator of increasing emphasis on standardized building methods, because the installation of a tiled roof requires that hundreds of elements should overlap correctly and fit snugly in order to ensure that the roof is stable and watertight.

In a recent study, Song et al. presented a comprehensive review of the roof tiles of the Loess Plateau in the Longshan Period which, although it provides key knowledge about early clay tiles in China, is mainly based on collections of roof tiles from archaeological field surveys. The conclusions presented therefore include several assumptions about composite-tile roofing methods^8^. However, we still have little idea about the level of standardization of clay tile production and in what manner the earliest composite tiles, in large numbers and with heavy loads, would have been manufactured, transported and finally placed to construct roofs. This limited understanding is primarily because: (a) samples of tiles from a single archaeological site are often small and most of roof tiles come from field surveys without clear archaeological contexts, (b) roofs often collapsed quickly after the building was abandoned and tiles are rarely found in situ, and (c) previous work on surviving tiles has been mainly descriptive. The recent discovery of a large number and variety of clay tiles in a single context during excavations at the Qiaocun site, which has since proven to be one of the earliest sites at which a large number of tiles has been recovered, provides an opportunity to examine early tile production and roofing methods in detail. Although most Qiaocun tiles are also fragments, a combination of 3D modeling and computer simulation has permitted the reconstruction of the tiles and of the roof they formed. Additionally, careful attention to historical documents and a re-evaluation of archaeological evidence from later periods have allowed for an elaborate reconstruction of composite-tile roofing techniques and their social/environmental motivations. This paper finds that these roof tiles excavated from the Qiaocun site on the Loess Plateau, dating to the early Longshan Period (~ 2400–2200 BCE) are the earliest composite tiles to date in the world (SI Appendix, Table S1).

## Qiaocun site and Qiaocun tiles

The Qiaocun site is located on the edge of the Loess Plateau at the headwaters of the Heihe and Daxihe tributaries of the Yellow River (Fig. 1B,C). Archaeological survey and excavation conducted from 2018 to 2021 by the Gansu Provincial Institute of Cultural Heritage & Archaeology and the School of Archaeology & Museology, Peking University, reveal that the site covers an area of ~ 103 hectares, making it the largest known Longshan settlement in the local river valleys within 2373 km^2^^11^. AMS radiocarbon dates indicate human activity on-site at ~ 2400–1900 BCE. This date range covers the whole Longshan Period (SI Appendix, Fig. S1, Table S1). The majority of settlements during this period appear to have been cave dwellings, a large number of which have been discovered along the Loess Hills. To date tiles have only been found on top of the Loess Plateau^12,13^ (Figs. 1C, 7D).

The tiles were adjacent to the contemporaneous structures at the site. The majority of the tiles recovered from the site were found deposited in a secondary context, a large ditch G2 (SI Appendix, Fig. S2). The extensive and complex use of the site in the Mid and Late Longshan Period means that the few Early Longshan structures that can be identified have been damaged by later activities^13^. Excavation at the Lushanmao, another Longshan site on the Loess Plateau, indicate that houses with thick rammed-earth walls were the predominant type of structure on top of the Loess Plateau^7^. The apparent deliberate destruction of tile-roofed buildings in the Qiaocin site in a single brief episode was confirmed by excavations that showed that the dated strata within the ditch were concentrated at ~ 2400–2200 BCE (Early Longshan) and belonged to the earliest phase of the site (SI Appendix, S1, Fig. S1, Table S1). Therefore, we believe the tiles unearthed from the ditch are very likely to come from one or more contemporaneous early structures which were of an large size.

**Figure 2:**
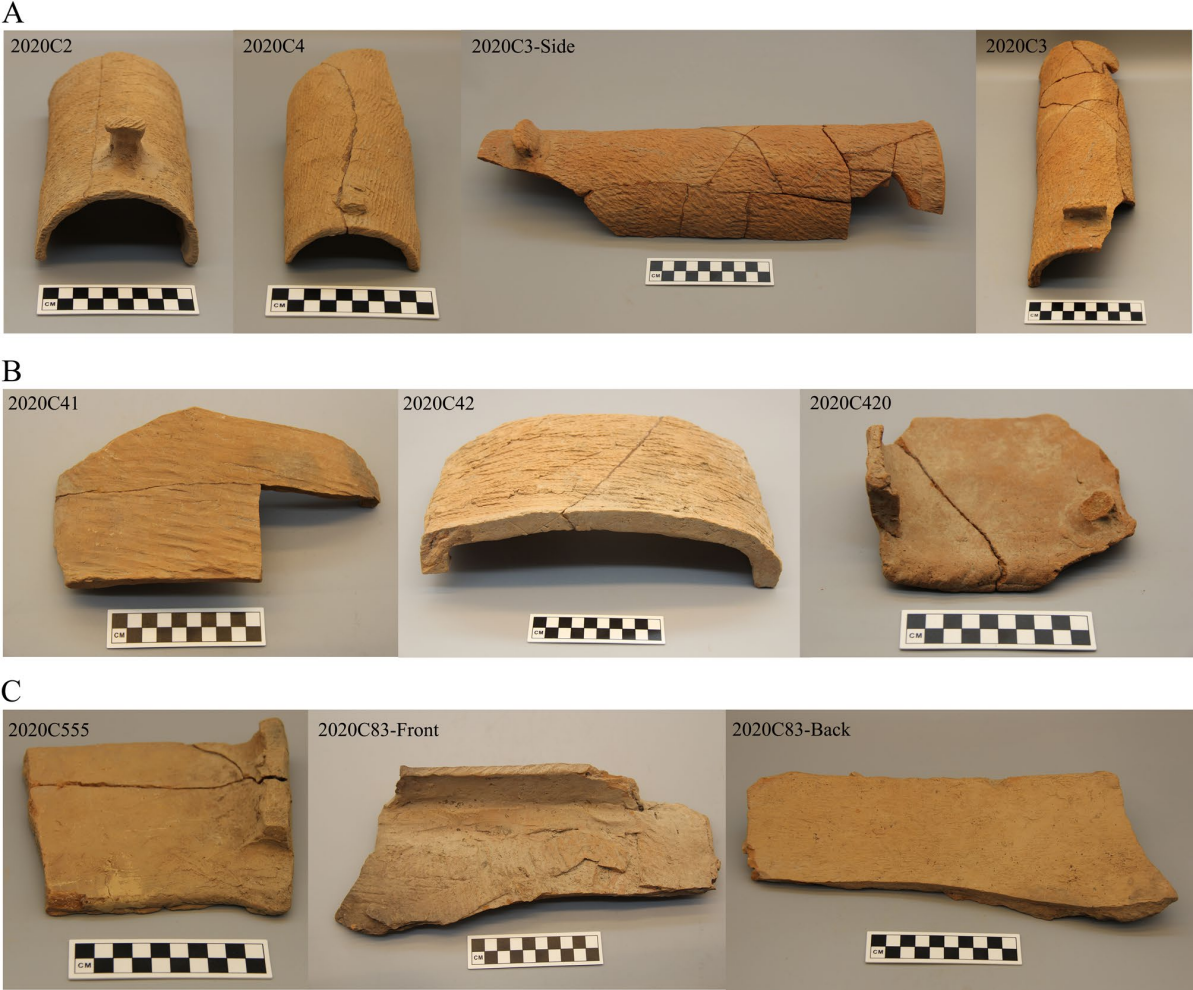
Examples of clay tiles from Qiaocun: (**A**) cover tiles; (**B**) pan tiles (2020C420 exhibits an end incision); (**C**) flat-pan tiles.

The tiles excavated from the Qiaocun are made of red fired clay with fine inclusions, which can be divided into two basic types: cover tiles and pan tiles. Cover tiles are semi-cylindrical with differently shaped ends: one end being narrower in width and lower in height and often with a clay nail (hereafter referred to as the “smaller end”), and the other end referred to as the “larger end” (Fig. 2A). Pan tiles are approximately trapezoidal, with raised sidewalls and also a distinction between the smaller and larger ends. Unlike the consistent shapes of cover tiles, pan tiles are found in three different sub-types (Fig. 2B): type a without nail or incision on end, such tiles have a slightly convex back, Type b with two incisions and a nail at the smaller end (these are of relatively low ubiquity compared to Type a), and Type c there is a greater uniformity in this tile type, these tiles are characterized by their flat back, and straight sidewalls-with-holes (Fig. 2C).

## Results

### Restoration of the tiled roof using historical/archaeological references, sherds statistics, and 3D modeling

The pan and cover tile system continues to be used throughout pre-dynastic and Imperial China. The *Yingzao Fashi*, a Chinese construction manual (1103 CE), outlines the procedures for installing composite-tiled roofs. Similarly, a comparison with tiles found at the Hetaoyuan site, a royal Buddhist temple dated to the Bei Qi dynasty (553–577 CE)^14,15^, allows for a reconstruction of the function of each tile types (SI Appendix, S2, Fig. S3). Although this historical text and the Hetaoyuan site are several thousands years from the Neolithic Qiaocun tiles, it is apparent that some rules and principles for the construction of composite-tiled roofs remained consistent over this time period. The reasons are as follows: (a) the form, style and ratios of cover and pan tiles are significantly similar, which indicates that installation techniques had not changed significantly in the interim (Table 1); (b) pan tiles at Qiaocun were on average twice as wide as the average width of the cover tiles (21.93 cm vs. 11.50 cm), this closely matches the dimensions described in the *Yingzao Fashi* and the tiles discovered at the Hetaoyuan site.

**Table 1:** Basic statistics of tile sherds from Qiaocun and Hetaoyuan.

	Cover tile	Pan tile	Flat-pan tile	Ridge tile	Total
Qiaocun	1760	2775	684	–	5219
Percentage	33.72%	53.17%	13.11%	–	100%
Hetaoyuan	2850	3587	–	1108	7545
Percentage	37.77%	47.54%	–	14.69%	100%

The ratios of the different types of tiles are fundamental to understanding how a particular roof was constructed. Most tile types are used on the roof surface, while a few are placed at specific locations, such as the eaves or ridge lines. In Qiaocun, cover tiles and pan tiles are the most commonly occurring, accounting for 33.7% and 53.2% of the tiles respectively. In contrast, the flat-pan tiles only account for 13.1% (Table 1). A similar ratio was observed at the Hetaoyuan site, suggesting similar roofing techniques.

Although specific tiles for roof ridges were recovered from the Hetaoyuan site^14^, no such tiles were recovered from Qiaocun. The smooth and flat morphology of flat-pan tiles seems better suited to being placed on a flat surface, such as on top of thick rammed earth walls. Traces of tiled roofs were observed during the excavation of rammed earth houses at Lushanmao, another similar, if slightly later Longshan site (~ 2300–2100 BCE) located on the northern Loess Plateau, about 250 km from Qiaocun (Fig. 1B)^7^. In addition, a Longshan house-shaped pottery vessel recovered from the Loess Plateau shows buildings with gable walls and double-pitched roofs (SI Appendix, S3, Fig. S4). Therefore, we suggest that the Qiaocun flat-pan tiles were intended to be attached to the tops of rammed-earth gable walls and then interlocked with the cover and other types of pan tiles which formed the roof properly.

Pan tiles with incisions at the ends formed the uppermost row of the roof with the incisions and nails oriented upwards toward the ridge. The form of this tile would help to anchor the tiles on the roof ridge. This means that the number of this tile is equal to the number of columns of pan tiles on a given roof. The proportion of eaves tiles recovered from the Hetaoyuan site, which from the historical period, varies from 8.3 to 33.5%. This is due to the variation in the sizes of roofs and tiles at the site^15^. In Qiaocun, pan tiles with end incision account for more than 19.46% (SI Appendix, Table S2A,B) of the tiles used, and since there are no specific ridge tiles in Qiaocun, we argue that in addition to sealing the gaps between rows of pan tiles, cover tiles were also used as ridge tiles.

**Figure 3:**
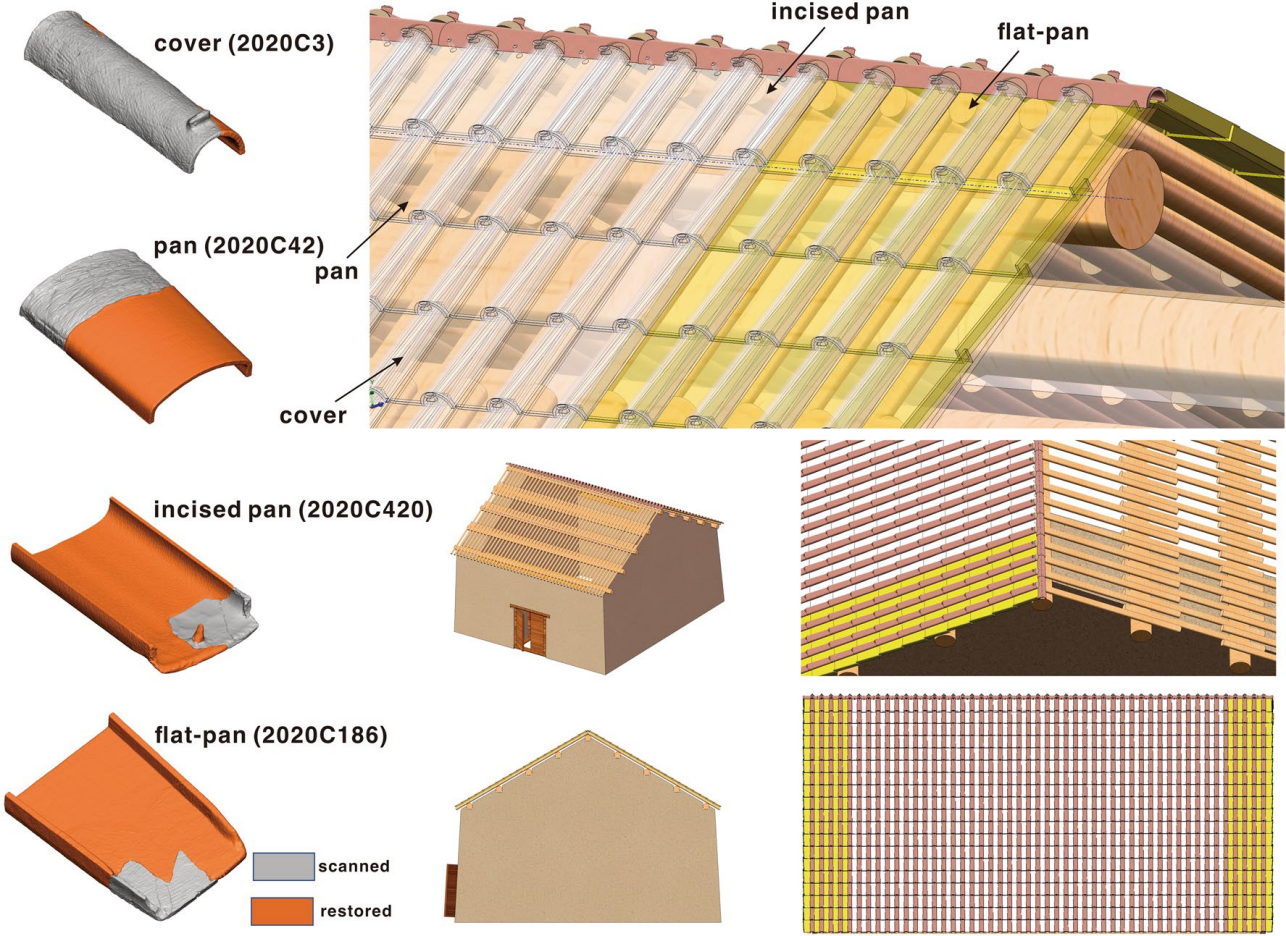
Restoration of Qiaocun composite tiles and tiled-roofing method.

Based on these inferences, we attempt to reconstruct a complete restoration of the tiled roof by 3D modeling using the most probable combination of pan and cover tiles (20 rows and 50 columns, see “Materials and methods”) (Fig. 3).

### Low-level standardization of Qiaocun tiles

**Figure 4:**
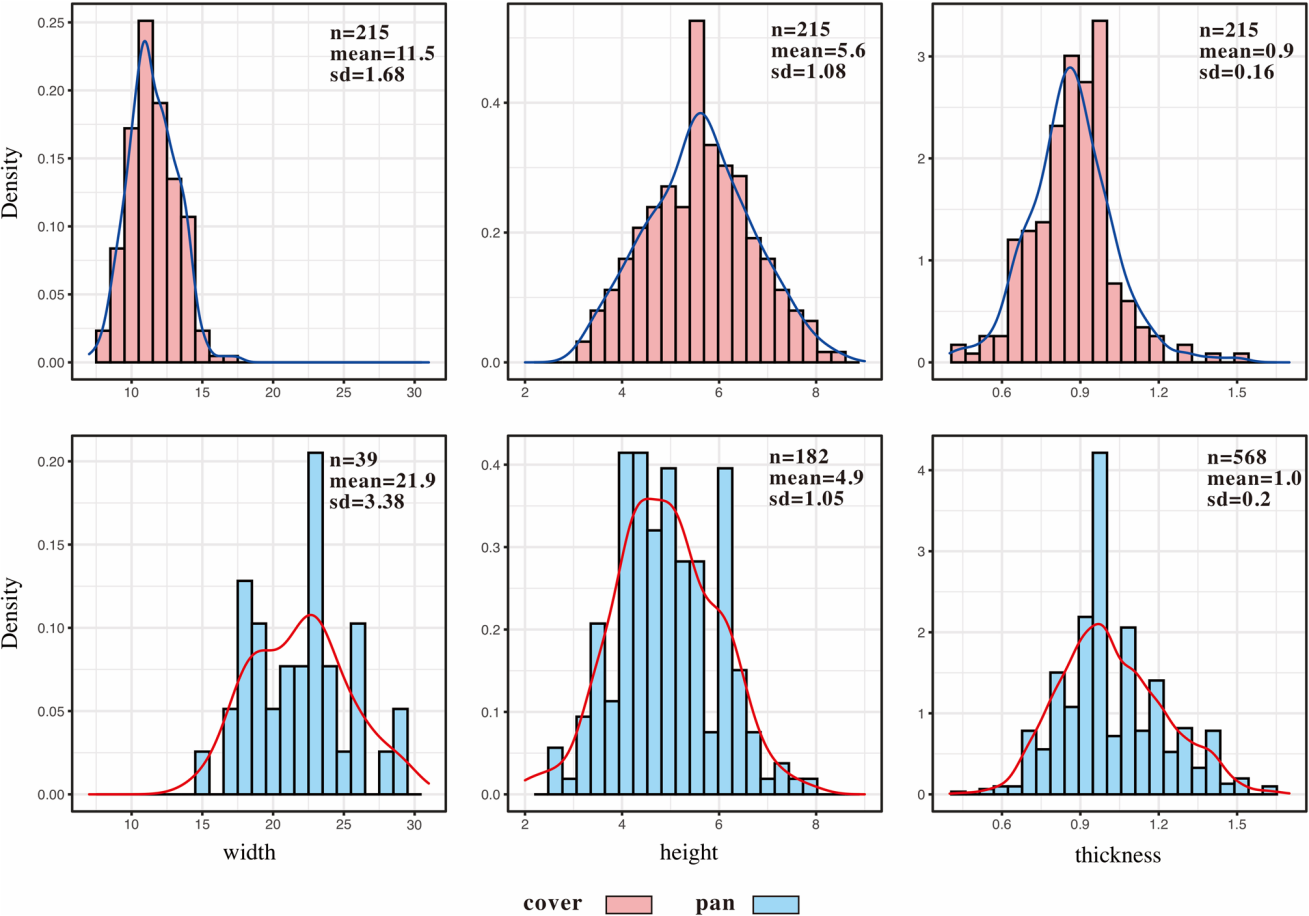
Dimensions of cover and pan tiles. All distributions are normal according to the Shapiro–Wilk test, with a 0.05 significance level.

The width, height and thickness of each tile fragment was measured and found to be normally distributed roughly (Dataset S1, Fig. 4). This suggests that the roof tiles recovered from Qiaocun were handmade without molds or similar tools (SI Appendix, S4). Coefficient of variation (hereafter CV) is a summary statistic used to calculate relative standardization in artifact dimensions, (see^16–19^), which can be further corrected via proposed by the Crown method (hereafter CV*)^18^ and others^20,21^ for the study of past pottery production. CV* values of ~ 10 to ~ 14% are indicative of specialist versus generalist production. Costin^16,22^, however, demonstrates the necessity of documenting the demand, function, and context of artisanal production before assuming that the relationship between standardization and specialization is clear. Qiaocun tiles of all types had CV* values of > 14%, but the CV* value of tile width is ~ 14%, indicating that a higher degree of manual control was exercised over the width than the height or thickness. In contrast, tiles from the Hetaoyuan site^14,15^, where pan and cover tiles of similar shape and size were found, had much smaller CV* values < 10%, indicating an increased degree of standardization in tile production in the intervening ~ 2000 years of Chinese history (SI Appendix, Table S3).

### Computer-based simulation indicating the significance of manual control for composite-tile roofing technique

For a roof to be functional, tiles of different shapes and sizes must be matched according to basic rules. This means that the vertical joins between pan-pan or cover-cover tiles and the horizontal joins between cover-pan tiles needed to match (see details in SI Appendix, S2, Fig. S9). Based on these rules, repeated sampling of tiles from the normal distribution of the morphological variables of each tile type were used to construct a roof using three simulation models (random, grouped, and manually controlled, see “Materials and methods”). These models were intended to evaluate the feasibility of the roofing process and the extent of intervention required on the part of the roofers given the observable variations in tile dimensions.

**Figure 5:**
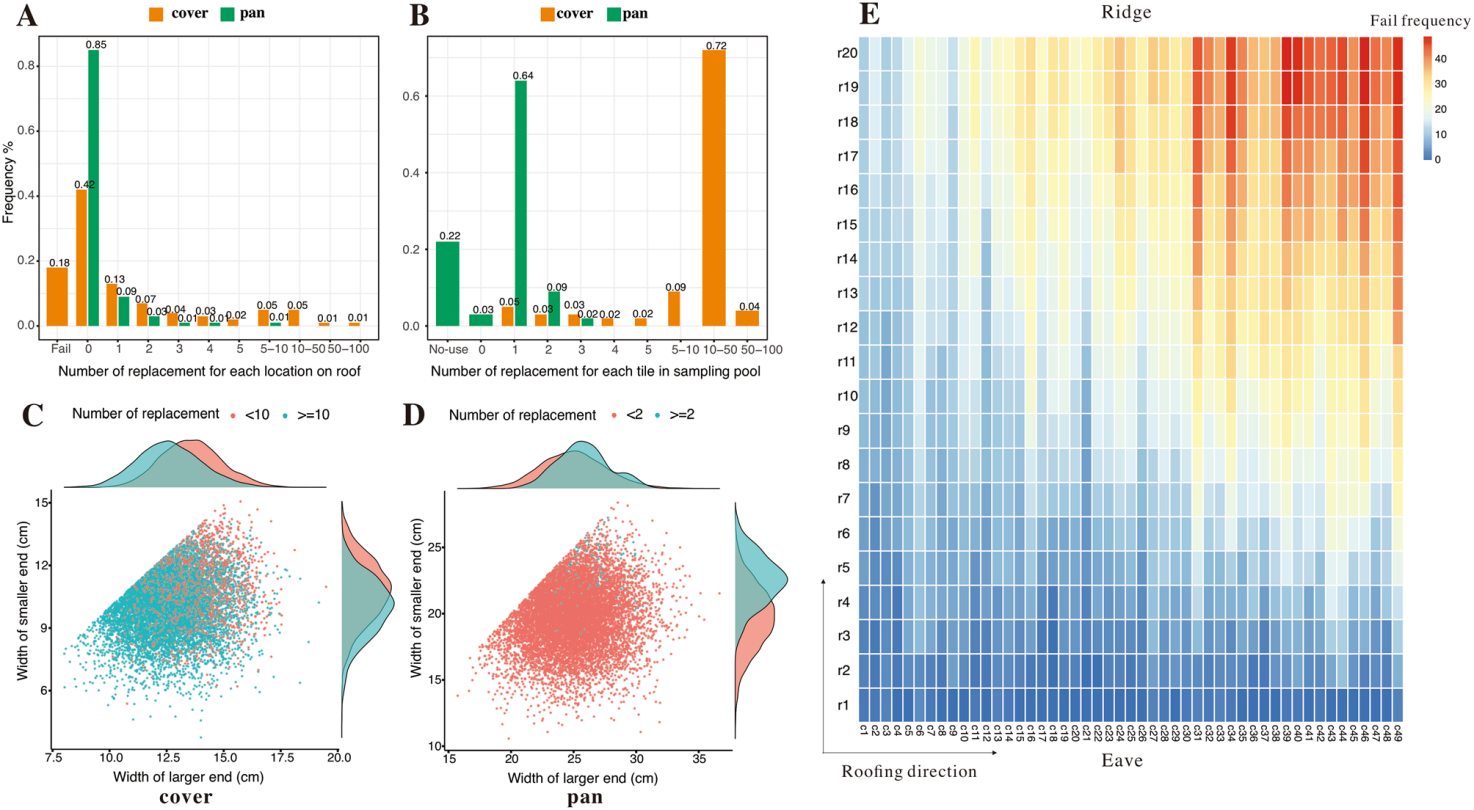
Statistics based on the random model. (**A**) frequency for the number of replacements for each location on roof; (**B**) frequency for the number of replacements for each tile in sampling pool; (**C**) plot of cover tiles on size in sampling pool with the number of replacements; (**D**) plot of pan tiles on size in sampling pool with the number of replacements; (**E**) heatmap for the failing frequency based on 100 random simulations on the roof (20 rows and 49 columns for cover tiles).

When running the random tile selection model, the design failed completely in 18 out of 100 simulations when RC (Reserve Coefficient, referring to the times for the number of tiles needed for roofing) is set to 1.5, as indicated by the failed tile installation as the last step of the roofing process (Fig. 5A). In unsuccessful simulations failure usually occurred in the second half of the process (Fig. 5E). Even when the simulations were successful, the frequencies of replacement for each location were often still too high to be acceptable. In all successful simulations, two percent of the locations were replaced more than 50 times to find a suitable cover tile (Fig. 5A). At the same time, over 4% of the cover tiles were selected and then rejected for installation on the roof over 50 times (Fig. 5B). This suggests that the variance in tile shape is too great for a random model to arbitrarily allow construction of a viable tiled roof. In examining the morphology of those tiles that were repeatedly rejected by the simulation, we found that these tiles were generally either pan tiles with too narrow larger ends or cover tiles with too wide smaller ends (Fig. 5C,D). This variance meant that the tiles could not connect correctly with the next tiles in the sequence.

**Figure 6:**
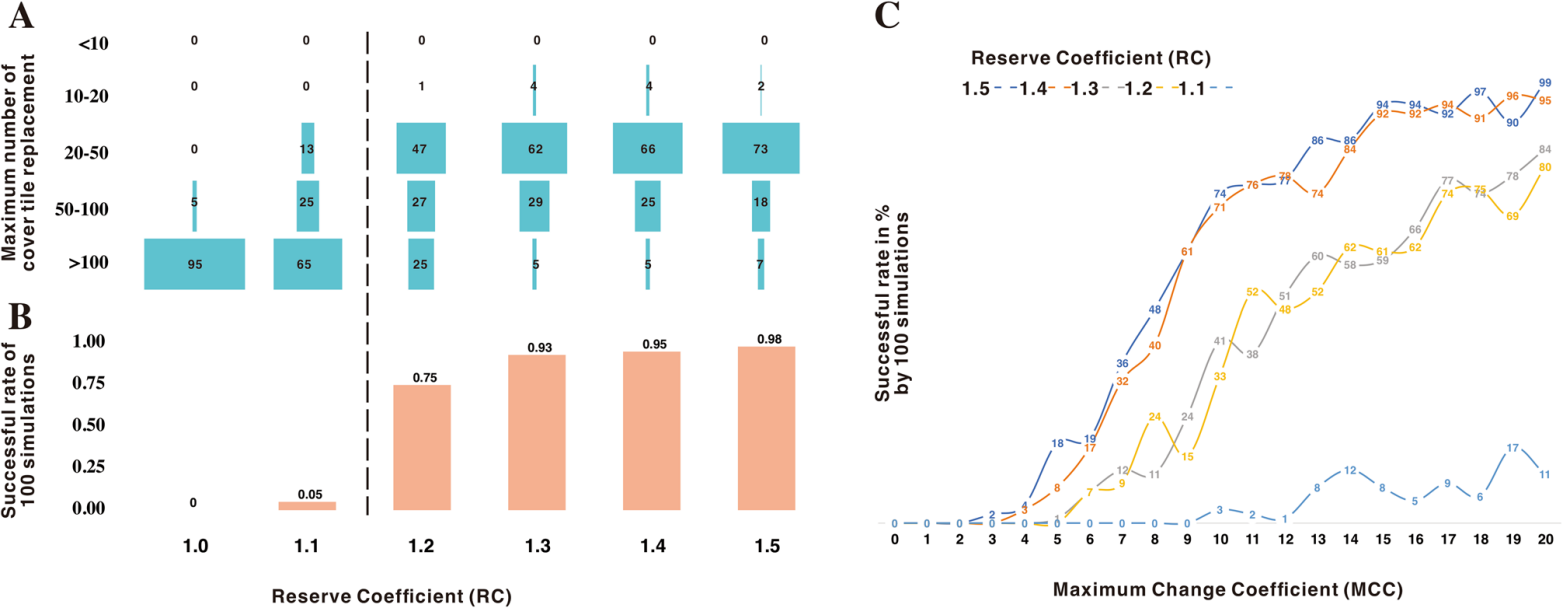
Results from the grouped and manually controlled models. (**A**) battleship plot of grouped model for the maximum number of cover tile replacements on the roof; (**B**) successful rate based on 100 simulations in grouped model by RC values from 1.0 to 1.5; (**C**) successful rate based on 100 simulations in manually controlled model by MCC values from 0 to 20 and RC values from 1.1 to 1.5.

After determining that a random model for tile selection was impractical, we ran a simulation using the grouped model. It is obvious that the total number of tiles in the sample pool has a significant impact on the results. When RC is greater than 1.2, the success rate of the entire project is over 75% and increases to over 90% when RC is 1.3. The frequency of tile replacement also decreased as the RC values increased (Fig. 6A,B). However, despite this improvement, the grouped model cannot completely solve the roofing problems. Even with a 90% success rate of simulations (RC >  = 1.3), there are still a large number of locations on the roof where more than 50 cover tiles (5–7% or 49–69 positions on average for each simulation, Fig. 6A) need to be tried before a matching tile is found, requiring some extreme component mismatches to be adjusted by hand.

Another manually controlled model was run. This model introduced a new parameter, MCC (Maximum Coefficient of Change, referring to the allowable maximum number of changes for one position on the roof), to prioritize the minimum change of tiles. What is remarkable about the results of this model is that the RC values still matter in the simulation and that a reserve of 1.2 times the tiles is essential for roofing success. When the RC values were greater than 1.4 and the MCC value was 10, the simulation had a relative success rate of 70%. When the MCC value was increased to 15, the success rate increased to 90% (Fig. 6C). The manually controlled model therefore appears to best reflect the process that would have been used to construct tiled roofs in Qiaocun.

Based on this analysis, manual control would have been a key factor for the successful construction of tiled roofs when dealing with low levels of standardization. In fact, this method is recorded in the *Yingzao Fashi* (SI Appendix, S2).

## Discussion

### The earliest known tile production within the context of environment and social complexity on the Longshan Loess Plateau

Both the CV* values based on measurements of tile frangments recovered from the Qiaocun site and computer-aided simulation indicate the clay tiles were likely produced by specialists. In comparison to many other products in the Longshan Period such as pottery^23,24^, jade^25^, stone tools^26^ and bone tools^27^ which show a high degree of standardization, these roof tiles were manufactured to a low-level standardization. This would then have meant that there was a requirement for frequent manual adjustment during the roofing process. According to the *Yingzao Fashi*, the production of tiles and the subsequent construction of tiled roofs required a huge investment in terms of labor (SI Appendix, S2). It is evident that production must have been a communal undertaking beyond the household level. It is therefore necessary to investigate the environmental and social-cultural contexts of Longshan Loess Plateau in order to understand how the tiled roofs with their high cost in terms of both materials and labor came to be constructed in this area at this time.

**Figure 7:**
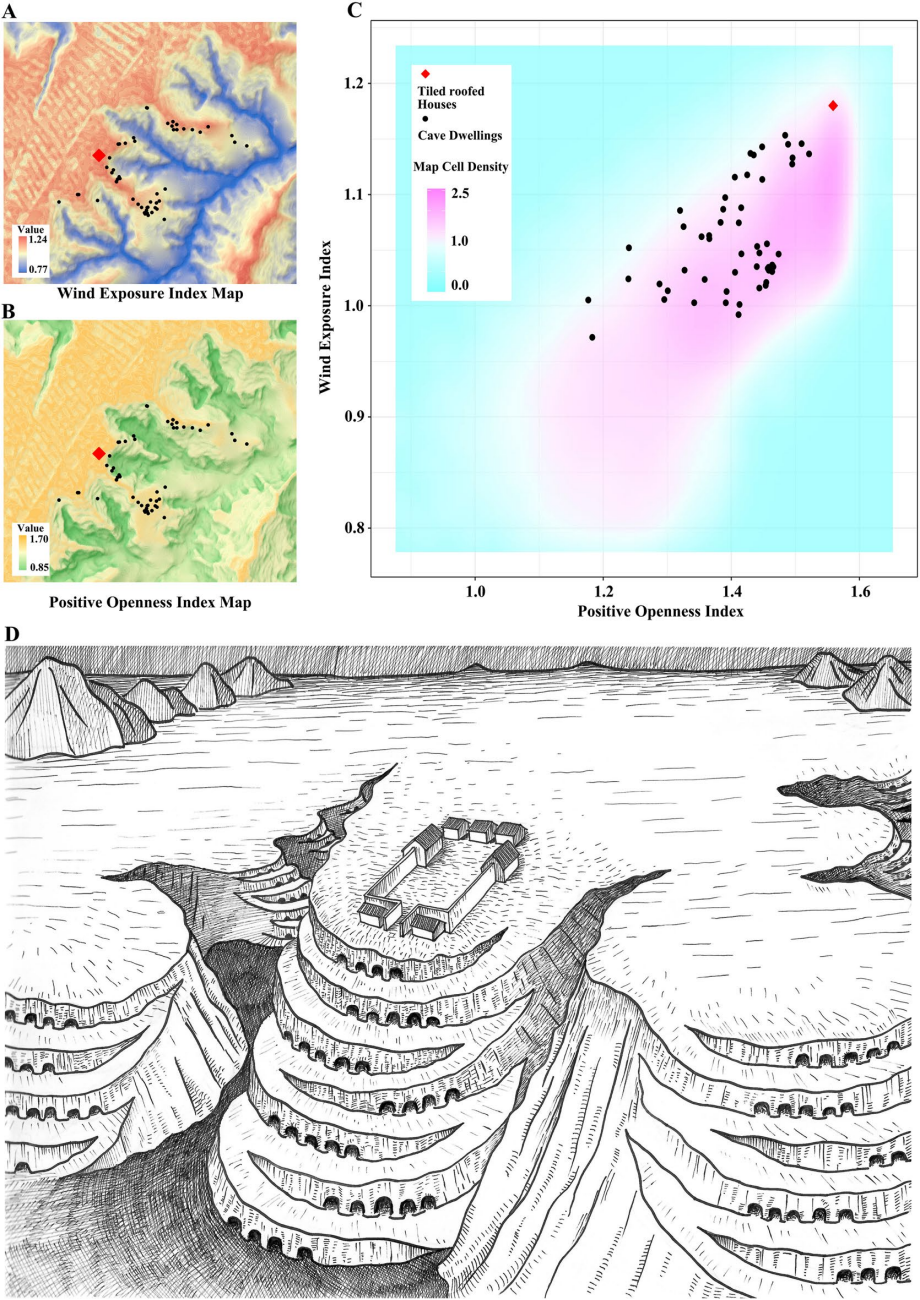
Locations of tile-roofed house and dwelling caves in Qiaocun. (**A**) locations of tiled-roofed house and dwelling caves on Wind Exposure Index Map; (**B**) locations of tiled-roofed house and dwelling caves on Positive Openness Index Map; (**C**) density plot of Map A and Map B with the locations of tiled-roofed house and dwelling caves; (**D**) sketch drawing of the landscape of the Qiaocun site with cave dwellings and tiled-roofed houses.

Firstly, the majority of Longshan structures are cave dwellings carved steep slopes of the Loess Plateau^28^. Such cave dwellings have limited prominence and visibility. Such locations were sub-optimal as venues for establishing major social connections (Fig. 7B,D). Therefore, elaborate large building complexes with tiled roofs were constructed on the Loess tablelands in order to, we believe, promote public cohesion. Such sites include Yingpanliang in Lushanmao^7^, Xiaoyuliang in Bicun^29,30^, and the tableland portion of Qiaocun^12,13^. However, the change of location from sheltered gullies to the top of Loess tableland meant that the structures were more exposed to the rain/hail (Fig. 7A,C). This environmental pressure may have led to the invention of clay tiled roofs to protect the exposed rammed earth walls of the buildings during this period.

The social context comes from the increasingly connected nature of the societies on the Chinese Loess Plateau. The social transitions which occurred between the Neolithic to Bronze Age in the Longshan Period have often been cited as integral to interactions between East and West in pre-historic times^31,32^. Previous studies of jades, bronzes, and other luxury objects indicate exchange or trade of goods between sites, indicating an enhancement in social interaction between settlements and regions^33,34^. Major centers on the Loess Plateau, such as Qiaocun, as well as Lushanmao (about 60 hectares) and Shimao (more than 100 hectares, about 460 km from Qiaocun, Fig. 1B), were involved in an active network of the social communication.

The excavation of Huangchengtai in Shimao unearthed various artifacts in the middens around the tile-roofed buildings, including bone jaw harps and needles in addition to off cut waste. Stone moulds for bronze casting, small bronzes, and the aforementioned jades were also found. This can be taken as evidence of the ritual ceremonies, feasting, on-site craft productions (bone needle making and bronze casting), and perhaps inter-community exchange^34^. We suggest that the tile-roofed houses served as a hub for these communal activities.

An increased emphasis on public social affairs on the Longshan Loess Plateau was accompanied by the introduction of new livestock and bronze into the region. The domestication of cattle and sheep in addition to the spread of bronze technology seems to have accelerated complex social transitions and the development of Bronze Age civilizations, not only in the West but also in the East^35–37^, including the Yellow River region of China^32^. Roof tiles are not the only architectural elements that started to appear during this period; clay pipes (cf. Laohuzui^38^, Taosi^39^, Pingliangtai^40,41^), which formed a part of the public drainage systems, have also been excavated. The invention of buildings with tiled roofs and pipes was undoubtedly related to the increasingly complex social/public affairs in the new settlement centers, which eventually fostered the growth of social management, collective power and the urban revolution, as noted by Gordon Childe^42^. Composite-tiled roofs were known to have been used continuously in public architecture from the ancestral temples of the Western Zhou onwards^43–46^. A tradition that can now be traced back to Qiaocun.

### Origin, development, and spread of composite-tile roofing techniques in East Asia

Based on present archaeological discoveries, the tiles recovered from Qiaocun on the Loess Plateau in China are the earliest example of composite clay tiles in the world (SI Appendix, Fig. S5). Although some earlier “tile” remains, which are presumed to be cover tiles, have been excavated from the Matengkong recently^47^, the composite clay tiles from Qiaocun are still the earliest assemblage to contain both pan and cover tiles (SI Appendix, S5). Tiles excavated from Longshan sites share the same tradition of composite-tile roofing techniques but show slight technological diversification between sites. For example, the composite tiles in Lushanmao (~ 2300–2100 BCE, SI Appendix, S5, Fig. S1) are mainly a combination of cover and flat-pan tiles, the cover tiles are crafted with mud strips along the edges perhaps as an innovation intended to allow greater control over overlap and fixing. These strips perform the same function as the nails on Qiaocun tiles. The presence of these different techniques indicates that the tiling technology advanced in different ways in these two locations (SI Appendix, Fig. S6). Song et al. have published another kind of flat-pan tiles in Qiaocun site from an archaeological survey; these tiles are specially designed with incisions at the smaller ends and shallow channels at the larger ends, as a tenon and mortise joint (SI Appendix, Fig. S7). The width of these flat-pan tiles can be divided into two groups (~ 15 cm and 5 cm), but no such tiles were found among the over 5000 tile fragments excavated from G2. Given the fact the predominance of later Longshan remains at the Qiaocun site, it is probable these flat-pan tiles belong to another more advanced tiling system. In summary, these differences suggest that there are various methods of manufacture and installation among the earliest known tiles of the Loess Plateau, and the roof tiles we excavated from Qiaocun are likely the most earliest form excavated to date (SI Appendix, Fig. S1, Table S1).

The invention of clay tiles was associated with an important change in building technology, the advent of thick rammed-earth walls. Rammed earth first appears as a construction technique in late Neolithic China at ~ 3000 BCE (cf.^48,49^), mainly in or near the Loess Plateau, and was initially used to create defensive walls around villages or early cities. Rammed earth walls have a far greater load-bearing capacity than the wattle-and-daub structures with thatched roofs which characterize the structures found in early Neolithic sites (SI Appendix, S6, Fig. S8). Thus, the invention of composite clay tiles and tile-roofing techniques which appears to have co-incided with the advent of rammed-earth walls can be viewed as the earliest such tradition in East Asia. This tradition appears to be distinct from the structures with stone or mudbricks walls and single tile roofs found in contemporary Greece (SI Appendix, S7).

From 1800 to 1046 BCE (Erlitou to Shang), there is a notable dearth of tiles from archaeological discoveries. Although some have been recovered from sites on the Loess Plateau or the along edges of the Loess Plateau, such as the Zaoshugounao site, belonging to the Proto-Zhou period in the Guanzhong Basin^50^ and the Sanxingdui site, in the Sichuan Basin^51^. However, there is no evidence of tile-roofed buildings at Erlitou or Shang sites. Both these cultures originated in the lower reaches of the Yellow River and its delta regions^52,53^ in eastern China. This further suggests that early tile-roofing techniques were limited to major sites on the Loess Plateau and the regions in close relations with it^54^.

During the Western Zhou (1046–771 BCE), the number of roof tiles recovered from archaeological contexts increases. The majority were found in sites in the Guanzhong Basin. The cover, pan, and flat-pan tiles are still present, however, there are two new forms: half tile-ends used to protect and decorate eaves and large cover tiles which were used as ridge tiles^45,46^. These specialized tiles indicate that a more formal roofing tile system was gradually being established. They had a more uniform shape, especially the pan tiles, which are now curved to third or quarter circles. The smaller ends of the cover tiles have a lip so that they could be more easily fitted into the larger ends of the next cover tile in the column, which in some cases the thickness of tile ends was also intentionally decreased^43,44^. Such a mortise-and-tenon style guaranteed the interlocking of pan–pan or cover–cover tile arrangements. In general, through to the end of the Western Zhou, composite clay tiles technology and structures built using thick rammed-earth walls appear to have been largely restricted to the Loess Plateau. This consistent use and evolution in tile-roofing techniques forms the “Longshan–Western Zhou tradition,” which can be paralleled with the “Greco-Etruscan tradition” in the West (SI Appendix, S7).

By the time of the Eastern Zhou (771–221 BCE), composite tiles had become widespread and are commonly found in archaeological sites throughout China. The development of timber frame buildings and the *dougong* method (SI Appendix, S8) during the Warring States Period (475–221 BCE) and the Qin–Han Dynasties (221 BCE–220 CE) meant that there was greater flexibility in the form of buildings with tiled roofs^55,56^ (SI Appendix, Table S5). It is during this period that molded tiles start to appear in the archaeological record, replacing the hand-shaped tiles of earlier periods^57^ (SI Appendix, S3). This shows the extent to which the shape, design, and manufacturing techniques for roof tiles had evolved in the end of the Eastern Zhou Period.

There is a shared tradition of composite-tile roofing technology across East Asia^58,59^. Archaeological discoveries demonstrate that the tiles and the associated roofing techniques were dispersed to the Korean Peninsula^60^, Japan^61,62^, the (now) Russian Far East^63^, and Southeast Asia^64,65^ from the third century CE onwards (Fig. 1A, SI Appendix, Table S4).

In summary, the earliest composite tiles and tile-roofing techniques in East Asia originated and developed on the Chinese Loess Plateau. The tiles were originally used to roof communal structures built using thick rammed-earth walls in the large sites (more than 100 ha) which formed regional centers. This gradually formed a Longshan–Western Zhou tradition for tile-roofed buildings. The composite-tile tradition spread from the Loess Plateau to the whole of China in the Eastern Zhou and became the most influential roofing technique across East Asia.

## Materials and methods

### The morphological measurement and statistic of tile samples

A total of 5219 clay tile fragments were collected from the ditch G2 by sieving with 10 mm mesh. The total weight of the collected fragments was 382.7 kg. All fragments were assembled to reconstruct intact tile patterns as much as possible. The fragments with morphological features were measured for width, length, height, thickness, and the expanded angle showing the slight taper between the larger and smaller end (Dataset S1).

CV* was then calculated according to the sample-size adjusted equation provided by Topi et al.^21^.

### The restoration of tiled roofs

We found samples that could be assembled from sherds and reconstructed into almost complete tiles. Based on these reconstructions, the length of the tiles can be estimated at ~ 40 cm, which roughly corresponds to the tiles found at Lushanmao (cf.^7^ 37.2–47 cm). The tiles are also of a similar height and width. The best-preserved tiles of each type from the Qiaocun excavation were scanned by high-resolution 3D scans with a Hexagon AtlaScan Max 3D scanner. The 3D models of these tiles were then virtually repaired to form complete and intact tiles and could be duplicated and combined into virtual composites using Geomagic Freeform and SolidWorks.

The size of the houses and roofs probably varied, however, thick walls would have been required to support half a tonne of composite to support the heavy tiled roof, the rammed-earth walls had to be thick enough (SI Appendix, S5, Fig. S8). Table S6 lists the most rational ratio of cover and pan tiles in numbers according to the different possible roof sizes. Since the pan tile has a larger surface area than cover tile (2.08 times larger when calculated from the 3D model in surface area), the percentage of pan tile sherds is relatively large compared to that of cover tile sherds. When the fracture rate of the cover and pan tiles is considered, all possible ratios of cover and pan tiles in sherds can be calculated simultaneously.

### The simulations

First, two coefficients are predefined in the simulation: a tolerance and a reserve. The tolerance refers to the extent to which two adjacent tiles can be connected. Even a small numerical deviation (e.g., a few mm) in the simulation can lead to the failure of the roofing process. In reality, however, this can be easily corrected by hand. In the simulation, we artificially set a tolerance of one standard error of the thickness. In the study, we relax the range of the RC value from 1 to 1.5 by 0.1, which corresponds to the times as many as tiles in the sample pools before roofing.

For the simulations, tile sizes are drawn randomly from a normal distribution of tile pattern dimensions, including width and thickness. For the larger and smaller ends, there are separate data sets for sampling cover tiles. In contrast, pan tiles are difficult to distinguish, because the sample size and sherds of the ends do not have unique diagnostic characteristics. Therefore, in this study, we use a bootstrap method to identify the larger and smaller ends from a pan tile width and thickness dataset (SI Appendix, S9, Table S7).

The basic rules for joining tiles refer to both longitudinal and transverse directions. In columns, the width of the outer dimension of the smaller end must be shorter than the inner dimension of the larger end of the previous or next tile. In rows, the cover tile should be able to cover the gap between two parallel pan tiles due to the difference between the larger and smaller ends (SI Appendix, Fig. S9).

$${Rule}_{pan}: \left\{\begin{array}{c}Success, if P\_{W}_{n+1}^{se}<P\_{W}_{n}^{li}\\ Failure, if P\_{W}_{n+1}^{se}\ge P\_{W}_{n}^{li}\end{array}\right.$$

$${Rule}_{cover}: \left\{\begin{array}{c}Success, if C\_{W}_{n+1}^{li}>C\_{W}_{n}^{se} and C\_{W}_{n}^{si}>(\left({P}_{left}{W}_{n+1}^{le}-{P}_{left}{W}_{n+1}^{si}\right)/2+\left({P}_{right}{W}_{n+1}^{le}-{P}_{right}{W}_{n+1}^{si}\right)/2)\\ Failure, if C\_{W}_{n+1}^{li}\le C\_{W}_{n}^{se} or C\_{W}_{n}^{si}\le \left(\left({P}_{left}{W}_{n+1}^{le}-{P}_{left}{W}_{n+1}^{si}\right)/2+\left({P}_{right}{W}_{n+1}^{le}-{P}_{right}{W}_{n+1}^{si}\right)/2\right)\end{array}\right.$$

Here, the *P_W* and *C_W* are the width of the pan and cover tiles and *n* (1 <  = *n* < 20) is the number of *n*-th locations on each roofing column. *l* and *s* are the larger and smaller ends, respectively, whereas *i* and *e* are the inner and exterior dimension of the ends, respectively.

The random model randomly selected tiles from the sample pools at each iteration. Any tile that could not be placed on the roof was again included in the sample pool. At the point where the simulation ran out of all possible remaining tiles in the sampling pool, the roofing simulation was considered to have failed.

The grouped model is based on the classification of variable width by one standard error (σ), with data exceeding two standard errors (2σ) eliminated. Therefore, for each end of the tile, there are four groups of data (− 2σ to − σ, − σ to 0, 0 to σ, σ to 2σ) and 16 groups in total, resulting in eight each for both the pan and the cover tiles in the sampling pool. Then when roofing adjacent tiles, the new adjacent tile is drawn from the same group with a higher priority (Dataset S2).

The manually controlled model is based on the grouped model, but introduces a parameter, MCC. When the change in tile size at a location exceeds the MCC value, the change process is temporarily stopped and the previous tile that caused the negative impact on the location is removed. This recall mechanism can be applied both longitudinally and laterally and can be considered as a practical adjustment made by the roofers in real time. If recall occurred twice, the entire roof simulation was defined as a failure.

## Supplementary Information

Supplementary Information 1.

Supplementary Information 2.

Supplementary Information 3.

Supplementary Information 4.

Supplementary Information 5.

Supplementary Information 6.

Supplementary Information 7.

Supplementary Information 8.

Supplementary Information 9.

Supplementary Information 10.

Supplementary Information 11.

Supplementary Information 12.

## Data Availability

All the data in this study are available in the supplementary materials. The codes for the simulations of tile roofing can be found on GitHub (https://github.com/haizhang0921/Qiaocun-Tiles).
