# Supplementary figures and images for: Reconstructing the earliest known composite-tiled roofs from the Chinese Loess Plateau
Source: Sci Rep. 2023 May 19;13:8163. doi: 10.1038/s41598-023-35299-x (PMC10199015; doi:10.1038/s41598-023-35299-x)

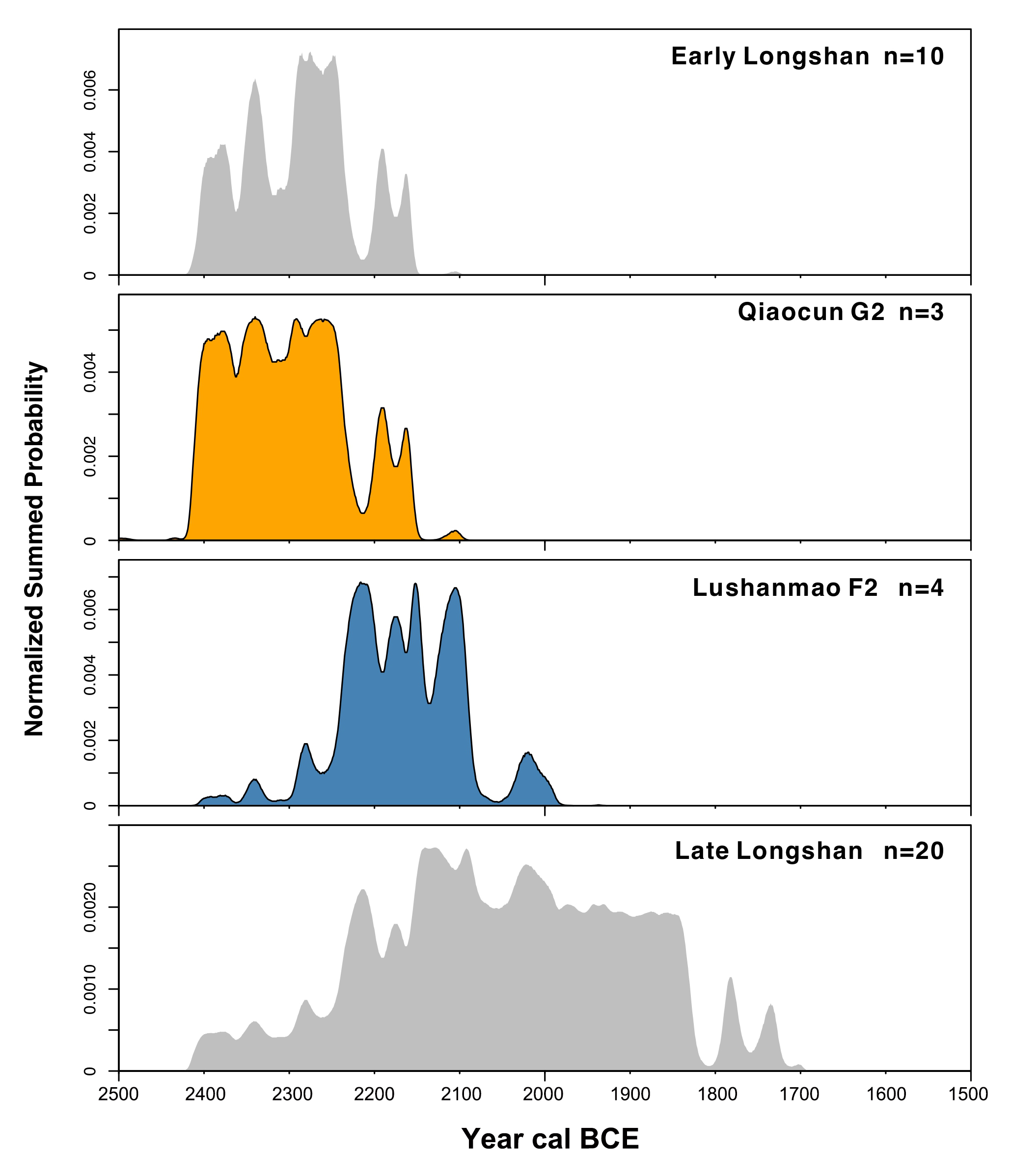

Supplement: Supplementary file 3 — Supplementary Information 3. [file 41598_2023_35299_MOESM3_ESM.jpg]

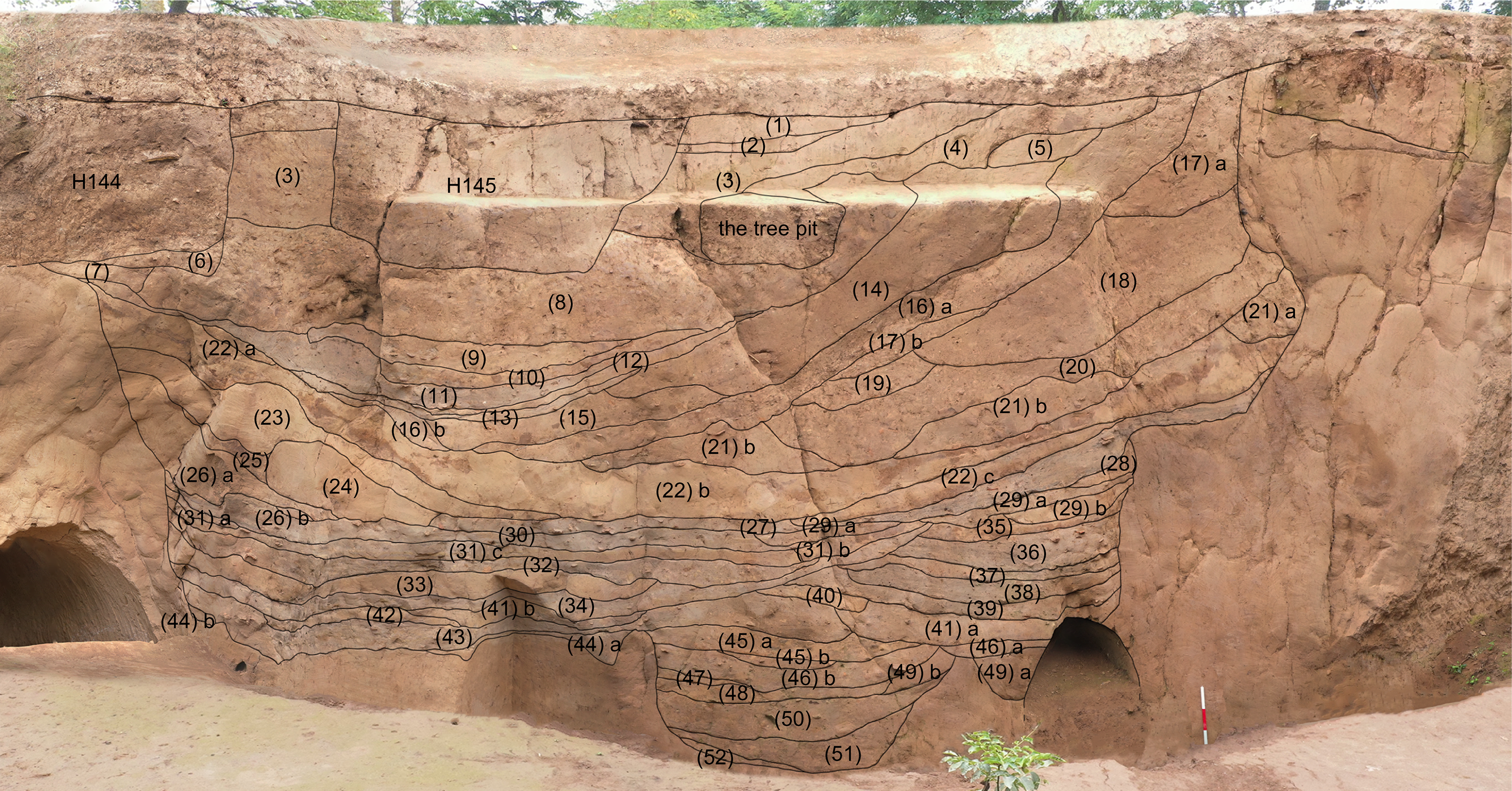

Supplement: Supplementary file 4 — Supplementary Information 4. [file 41598_2023_35299_MOESM4_ESM.tif]

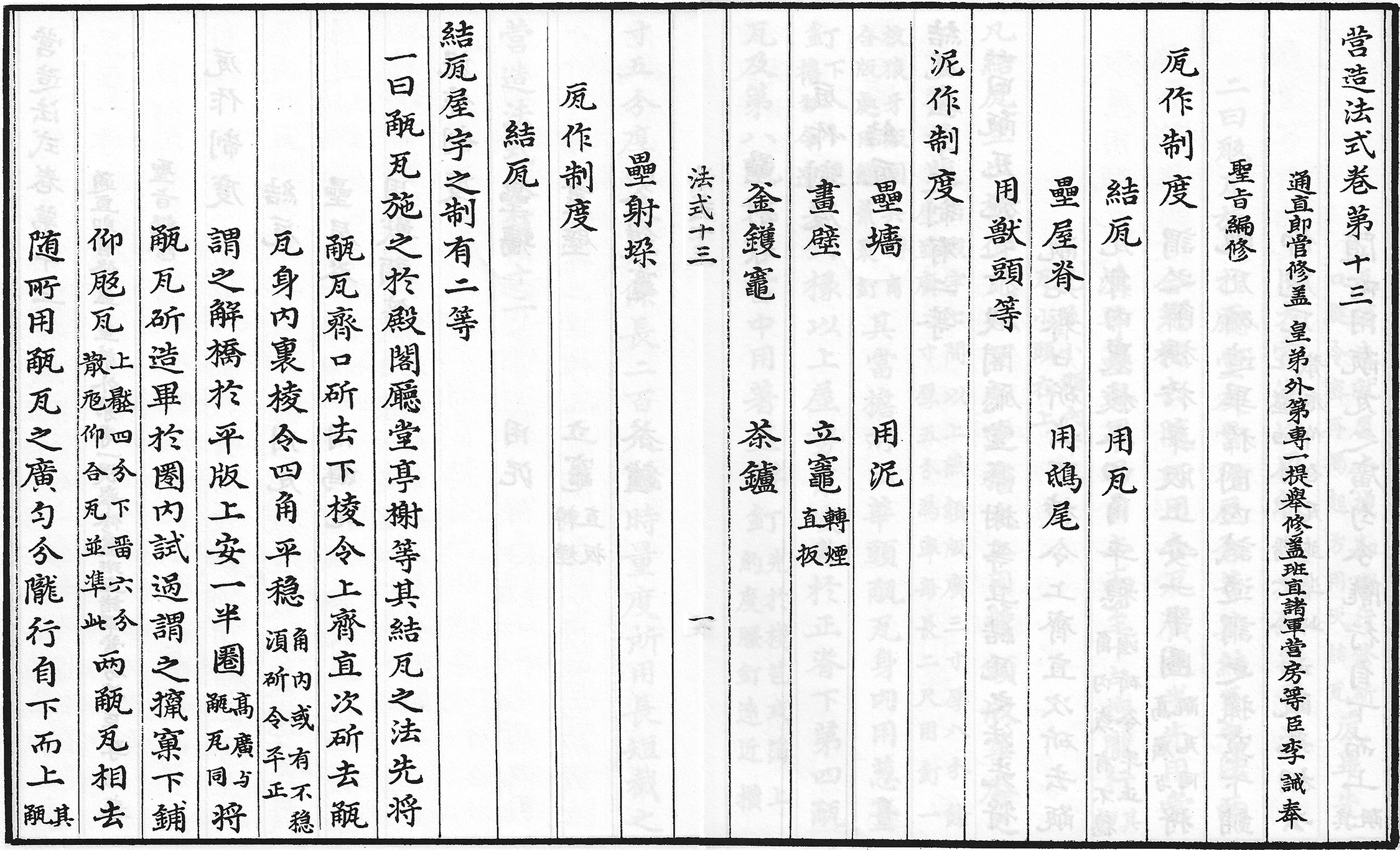

Supplement: Supplementary file 5 — Supplementary Information 5. [file 41598_2023_35299_MOESM5_ESM.tif]

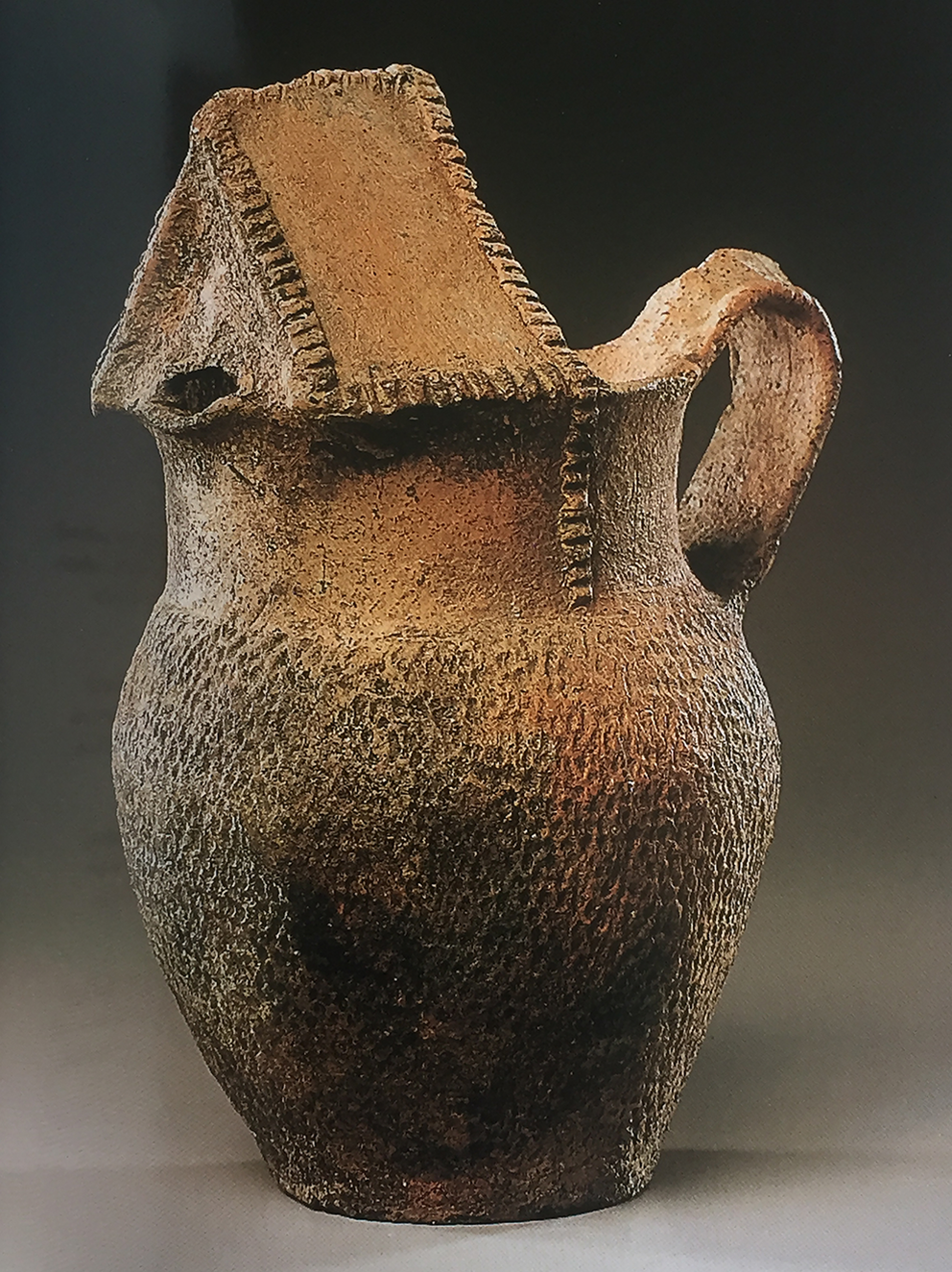

Supplement: Supplementary file 6 — Supplementary Information 6. [file 41598_2023_35299_MOESM6_ESM.tif]

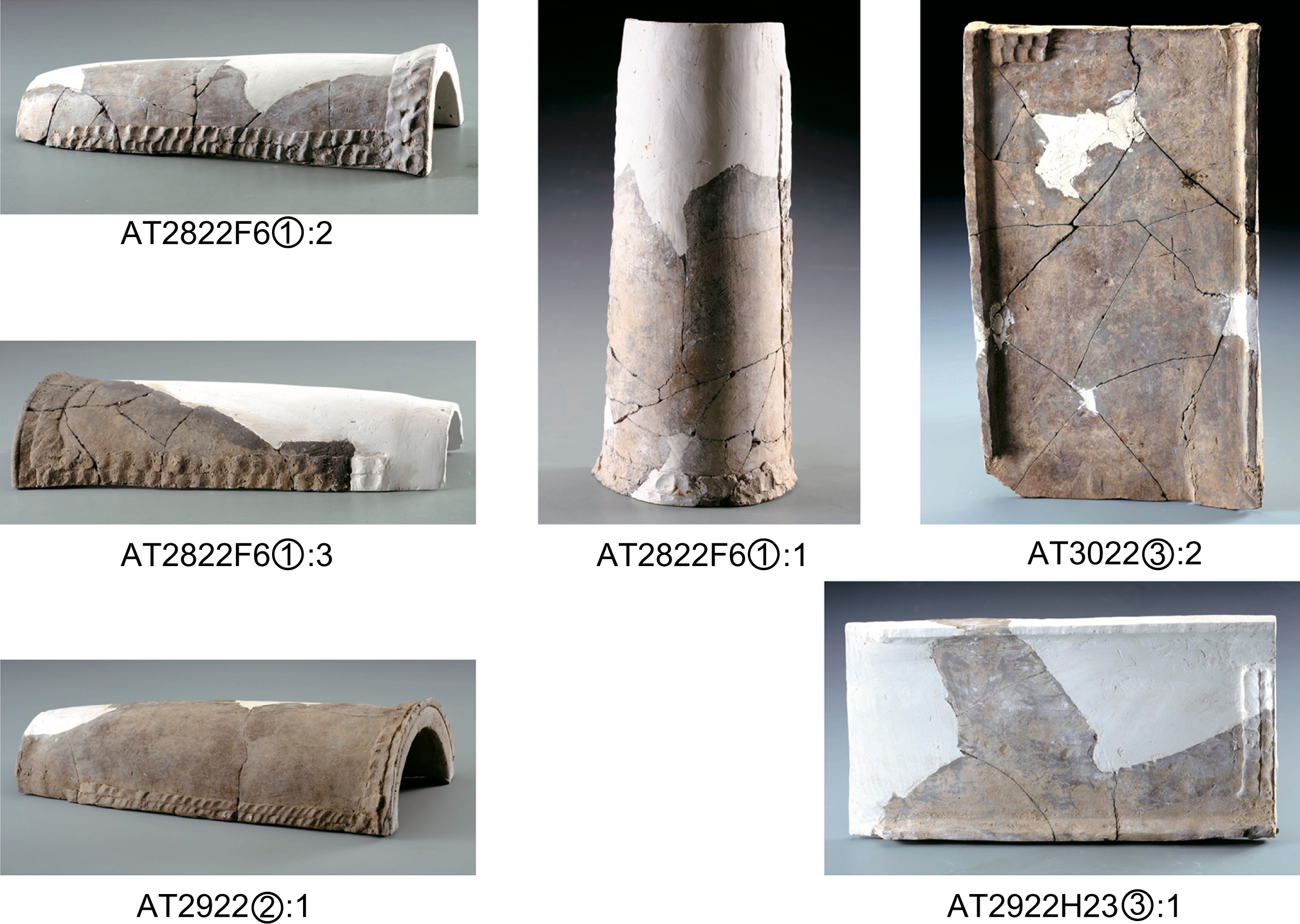

Supplement: Supplementary file 7 — Supplementary Information 7. [file 41598_2023_35299_MOESM7_ESM.tif]

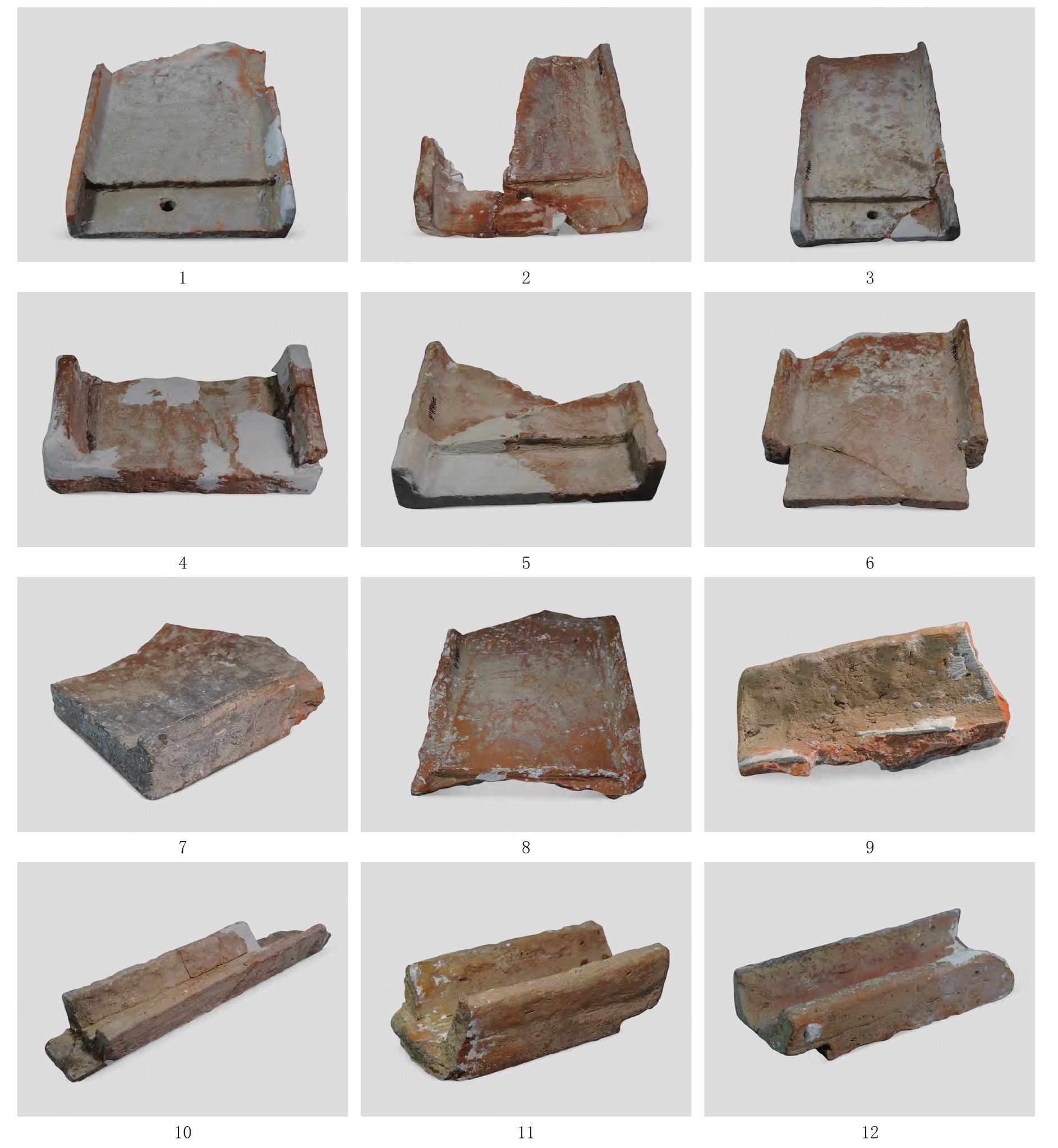

Supplement: Supplementary file 8 — Supplementary Information 8. [file 41598_2023_35299_MOESM8_ESM.jpg]

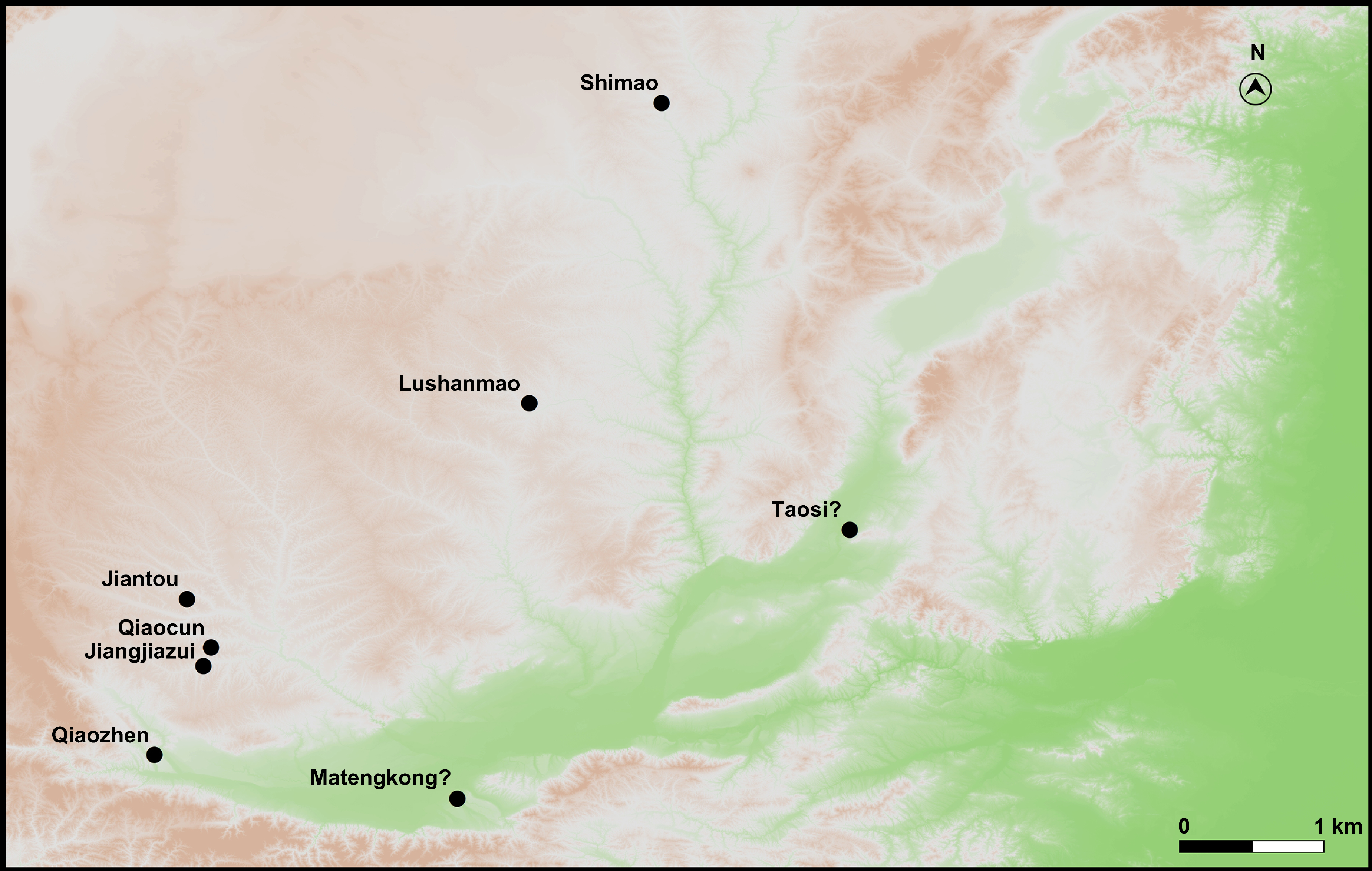

Supplement: Supplementary file 9 — Supplementary Information 9. [file 41598_2023_35299_MOESM9_ESM.tif]

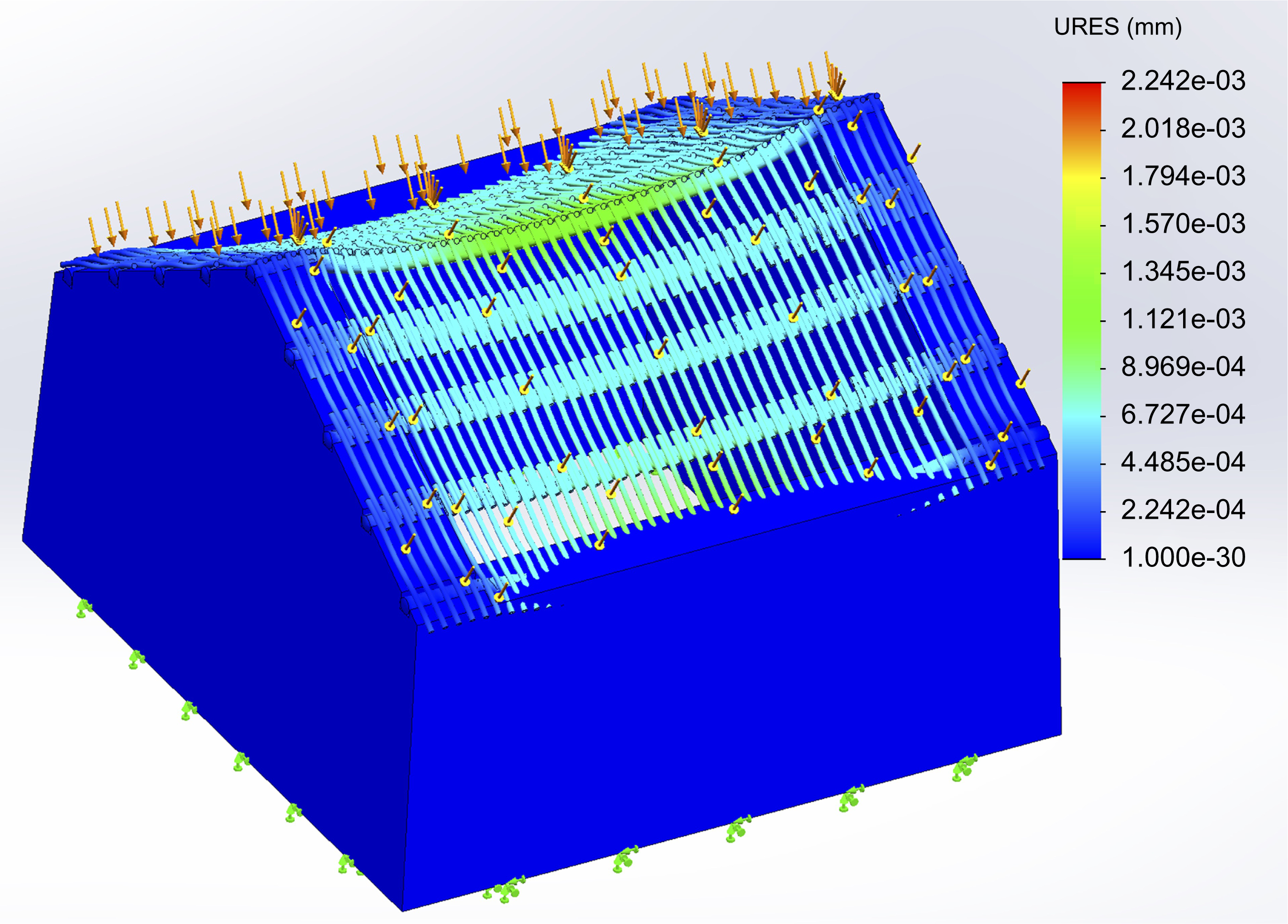

Supplement: Supplementary file 10 — Supplementary Information 10. [file 41598_2023_35299_MOESM10_ESM.tif]

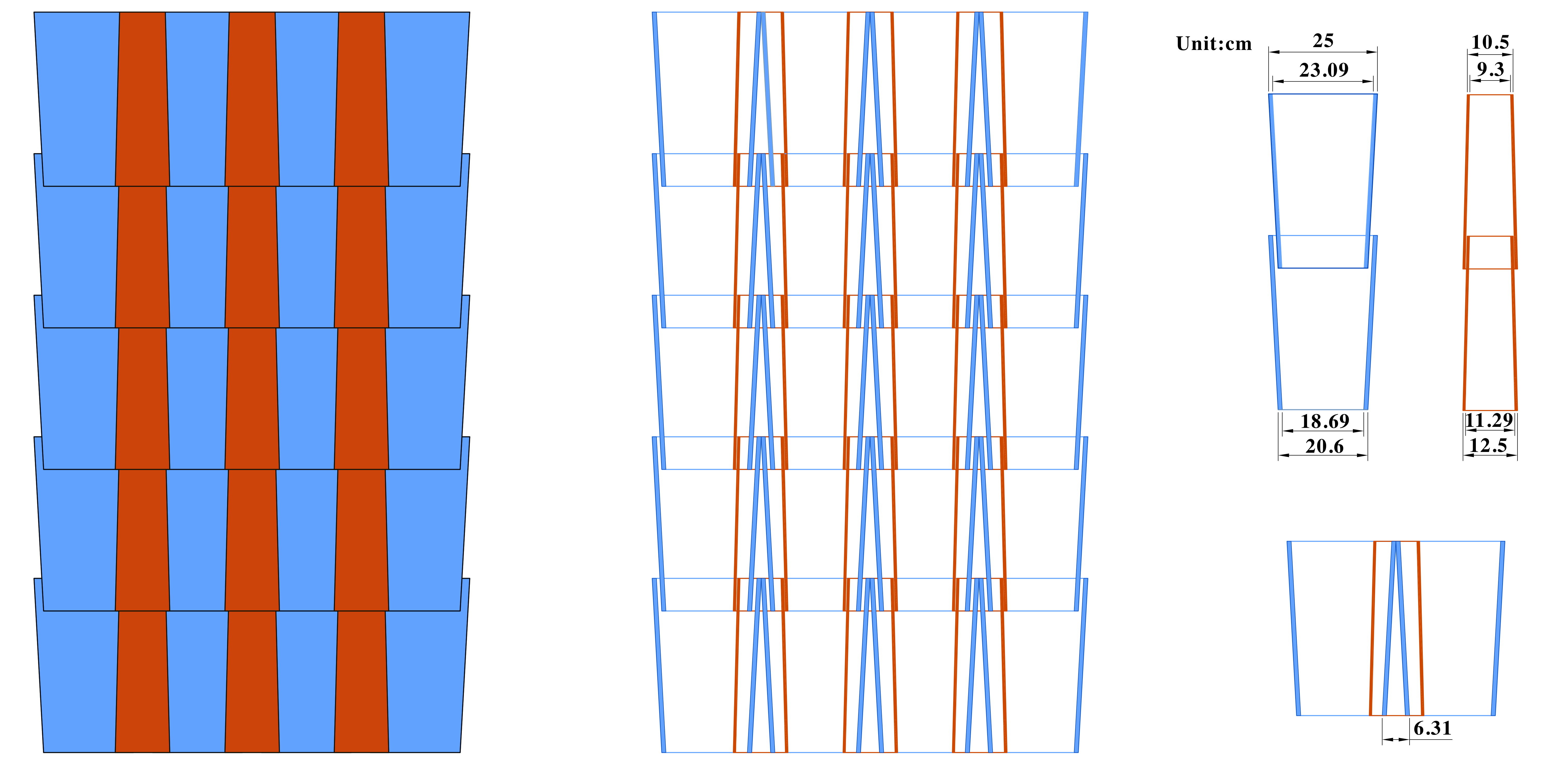

Supplement: Supplementary file 11 — Supplementary Information 11. [file 41598_2023_35299_MOESM11_ESM.jpg]
