# Supplementary material for: Reconstructing the earliest known composite-tiled roofs from the Chinese Loess Plateau
Source: Sci Rep. 2023 May 19;13:8163. doi: 10.1038/s41598-023-35299-x (PMC10199015; doi:10.1038/s41598-023-35299-x)
Supplement: Supplementary file 12 — Supplementary Information 12. [file 41598_2023_35299_MOESM12_ESM.docx]

**Supporting Information for**

Reconstructing the earliest known composite-tiled roofs from the Chinese Loess Plateau

Yijing Xu^1†^, Jing Zhou^2†^, Jianlong Zhao^2^, Guoke Chen^2^, Wen Li^2^, Mingzhi Ma^3^, Francesca Monteith^4^, Shengyu Liu^1^*, Minghao Peng^1^*, Andrew Bevan^5^*, Hai Zhang^1^*

1. School of Archaeology and Museology, Peking University, 5 Summer Palace Road, Beijing 100871, China;
2. Gansu Provincial Institute of Cultural Relics and Archaeology, 165 Heping Road, Lanzhou 730000, China;
3. Shaanxi Academy of Archaeology, 31 Leyou Road, Xi’an 710054, China;
4. School of Cultural Heritage, Northwest University, 1 Xuefu Street, Xi’an 710127, China;
5. Institute of Archaeology, University College London, 31-34 Gordon Square, London WC1H 0PY , United Kingdom.

* Shengyu Liu, Minghao Peng, Andrew Bevan, Hai Zhang.

Email: liushengyu@pku.edu.cn; 1706186129@pku.edu.cn; a.bevan@ucl.ac.uk; haizhang@pku.edu.cn

**This PDF file includes:**

Supplemental Information S1 to S9

Figures S1 to S9

Tables S1 to S7

**Other supplementary materials for this manuscript include the following:**

Datasets S1 to S2

**S1. Further research on the Qiaocun site and Qiaocun tiles**

The archaeological report has mainly introduced our preliminary work, especially at dense pits in the southeast plateau of Qiaocun [1]. Here we would like to provide further information on the Qiaocun site and Qiaocun tiles.

**Ditch G2 at the Qiaocun site**

Ditch G2 (Fig. S2) was located at the southeast edge of the plateau, where the elevation is 1.8–2 m lower than the main excavation area. Stratified below level one, the ditch was 10.1 m in diameter at the top, 1.95 m at the bottom, and 6.75 m in depth. The fillings in the ditch were divided into 52 sub-layers, containing pottery, animal bones, and numerous building material remains, like roof tiles, fragments of the lime-plastered floor, and burnt soil, at each level.

According to the fieldwork, dense pits including square pits and ash pits destroyed the earlier rammed-earth foundation and other architectural remains, which were closely associated with building material remains including roof tiles found at the ditch G2. Therefore, the ditch and its containing belonged to the earliest phase of the site.

**Roof tiles in the ditch G2**

Archaeological investigation and excavation suggested that roof tiles and other building material remains were mostly found in this ditch instead of the plateau. Besides these, most tile fragments were highly broken and torn down. We suspect that tile-roofed buildings suffered deliberate destruction and that building materials including numerous roof tiles were abandoned and moved to this ditch.

To determine the dates of roof tiles, we collected animal bones and teeth from levels 10, 30, and 40 for AMS dating. The results were concentrated on ~2400–2200 BCE (Fig. S1, Table S1), suggesting that the deposits were formed in a short time at about 2300 BCE. In addition, we also attempt to gather the newest radiocarbon dates of sites in the Longshan Period to confirm roof tiles’ location in chronological sequence (Fig. S1, Table S1). Based on the latest evidence, we prefer to conclude that roof tiles at Qiaocun dated to the early Longshan Period.

**S2.** **Historical and archaeological references**

Roof tiles continued to be used throughout Imperial China. Though most rules and principles of tile production and installation were first systematically documented in the *Yingzao Fashi*, edited by Li Jie and published by the Northern Song sovereign in 1103 CE, we believe that they were established much earlier through common practice and remained consistent.

We observe that some basic features of the roof tile system remain consistent. In addition, another site called Sanyangzhuang, a settlement of the late Western Han (~50 BCE–8 CE) where tiled roofs remained in situ and intact, suggested that their main tile types and the rules of installation conformed to the record in the *Yingzao Fashi*, appearing a thousand years later [2]. Therefore, we believe that citing the research on tile remains at Hetaoyuan and the document in the *Yingzao Fashi* as references is sound.

**The *Yingzao Fashi***

There are 34 chapters in the *Yingzao Fashi*, including five parts arranged systematically: basic terms, regulations, labor work, materials, and drawings.

Tile types and rules of tile imbrication

The 13^th^ chapter prescribes the standards and regulations for clay tiles, which contain tile types, relevant rules for the longitudinal and horizontal joints, and hierarchy for the usings of tiles and architecture (Fig. S3).

Regarding the rules and principles of tile installation, in general, it was assumed that the pan tiles were first attached to the roof structure with the concave surfaces facing upward, with the cover tiles placed over the gap between two pan tiles to create a watertight roof. For the longitudinal joint of pan tiles, the ratio of overlap between adjacent tiles was established. For the horizontal joint of cover-pan tiles, it is suggested that the distance between two adjacent cover tiles is equal to their width, which also means that the width of the pan tiles is about twice that of the cover tiles.

Based on our observations about tiles at the Qiaocun site, the presence of nails on the convex sides of the cover tiles to prevent slippage and the fact that the larger and smaller ends of the tiles fit together both horizontally and longitudinally indicate that the smaller ends of the cover tiles were attached upward toward the ridges. In contrast, the pan tiles, with smaller ends, were placed facing downward to the eaves. The average width of the pan tiles was twice that of the cover tiles, which is particularly consistent with the records.

Furthermore, the *Yingzao Fashi* describes the detailed process of roofing. The tiles are slightly struck to remove the irregular part and then placed on a standard half cylinder to select tiles of a uniform size. They are arranged, and the joints are checked on the ground before they are assigned and fixed on the roof.

This indicates that the manufacturing technology of the tiles in 1103 CE was not yet mature, and the tiles were divided into subgroups and tested on the ground in advance. Therefore, when designing the simulation models, especially the grouped model and manually controlled model, we carefully consider the importance of manual control (see Materials and Methods).

Labor investment in producing the roof

The *Yingzao Fashi* also provides information to estimate labor investment in person-hours needed to produce one roof. It is measured in gong*功*, a day’s work for one skilled laborer. In this research, the tiled roof, 20 rows by 50 columns, would need at least 1960 cover tiles and 2000 pan tiles with a length of 37 cm, and making and finishing these tiles cost about 83.03 gongs. It is also worth noting that tile making needs rather specialized artisans. Tile roofing would require an additional 27.28 gongs. In addition, the cost of supporting labor such as transportation during roofing should be considered, and this book suggests that this kind of cost is calculated as twice of that of the main labor, which in this study would equal 54.56 gongs. Therefore, a tiled roof of this size in the Northern Song Dynasty (960–1127 CE) would have meant at least 165 days of labor for a skilled worker, as shown in the records in the *Yingzao Fashi*.

However, technical progress in the production of tiles, full-time specialists, etc. made the construction of a tiled roof in the Song Dynasty much cheaper and easier than in the prehistoric period.

**Hetaoyuan site**

The site of Hetaoyuan, a royal Buddhist temple of the Beiqi dynasty (553–577 CE), retained numerous roof tiles. From 2015 to 2017, Peng conducted morphological measurement and collected statistics on these tile fragments, and the primary research allows a reconstruction of the function of these tile types [3].

In this study, we carefully compare the materials from the Hetaoyuan and Qiaocun sites and attempt to understand the structural function of particular tiles.

**S3.** Building with a double-pitched roof

The Lushanmao site is situated on the northern Loess Plateau in the upper branches of the Yellow River and also belongs to the “arc” [4]. Archaeological investigations and excavations in 2014 and 2016–2018 have revealed that the site dates from ~2300–2100 BCE and covers an area of about 200 ha. The quantities of jade, the large building complexes and the vast areas indicate that the Lushanmao site was the central settlement of the local society [5]. At this site, the large rammed-earth buildings were built only on the hills of the Loess Plateau, while the cave dwellings were widely spread on the steep slopes of the loess. Therefore, the rammed-earth buildings of Lushanmao and Qiaocun had many identical characteristics, and we believe that the rammed-earth buildings of Lushanmao may be a reference for the large buildings destroyed at Qiaocun.

Archaeological excavations in Lushanmao in 2016–2018 focused on a site called Dayingpanliang and found a foundation of rammed earth with three groups of courtyard-style buildings. The No. 1 group of buildings occupies the northern part of the hilltop, consisting of three main buildings and several dependent ones. The individual area of the main buildings is calculated to be 175–225 m^2^. To the south of these courtyard-like constructions are the groups of buildings No. 2 and No. 3, whose individual buildings cover an area of 60–90 m^2^. Of the above buildings, the report has published the basic information on building 5. It is 13.32 m long and 11.21 m wide; however, the front part of the foundation is slightly destroyed, and the original area may be larger. This house consists of rammed-earth walls with a residual height of 0.41–0.62 m, a lower width of 1.8–2 m and an upper width of 1.31–1.55 m. In addition, there is no evidence of timbering. We believe that it is a tiled roof, as clay tiles were discovered on the floor and the collapsed remains. Building 5 shows that the thick walls themselves should support the tiled roof, and long roof beams over which the pitched roof should be placed must be supported on two side walls. Therefore, the roof of the house should be a double-pitched-roof style.

A jug from the Qijia culture (~2500–1600 BCE) exhibited in the Asian Art Museum of San Francisco could also serve as an important reference (Fig. S4). The jug is made of earthenware with sculpted decoration and has a height of 22.8 cm and a diameter of 12.4 cm. In particular, it is decorated with a roof-shaped attachment in a double-pitched-roof style. As for the clay spirals, we believe that this is a decoration that was common in the potteries of the Qijia culture, and not an imitation of roof tiles.

**S4.** The manufacturing technique of roof tiles

We infer tiles’ production techniques according to the traces of manufacturing on the concave surface of tiles. To make one cover tile, the tile-maker prepared clay coils and raised them to form a conical tube; then, the tube was split in half by wire or slice cutting. We believe that no mold was used, because the tiles’ ratios of height to width were various. As for pan tiles, they were also handmade. Most were similar to cover tiles, while some flat-pan tiles were formed with slices of clay.

Tile-making techniques were extremely likely inspired by pottery. Archaeologists have conducted research on pottery dating from the Miaodigou II (the Early Longshan Period) to the late Longshan Period, discovered in its neighbors like southern Ningxia and southern Shanxi [6, 7], and there appears to be a consensus that hand coiling was dominant in pottery-making.

We also noticed that Song et al.’s paper talked about the manufacturing method of another kind of flat-pan tiles [8]. We believe their statement that the above flat-pan tiles were formed with scattered or intact slices of clay by hand is sound.

**S5.** Review of clay tiles in prehistorical China

Clay tiles were first invented in the Chinese Loess Plateau around 2500 BCE. In recent years, archaeologists have found various tiles at the sites in Shaanxi and Gansu Provinces (Fig. S5).

**Tiles found in prehistoric China**

First of all, we here add an introduction about another kind of flat-pan tiles of **Qiaocun** (~2500–1800 BCE) published by Song et al [8]: (a) these tiles are specially designed with incisions at the smaller ends and shallow channels at the larger ends, as a tenon-and-mortise joint, (b) according to width, they can be divided into two groups, one about 15 cm wide and the other about 5 cm, (c) no similar tile is found among the 5219 fragments of our material. We, therefore, suspect that these flat-pan tiles belong to another more advanced system (Fig. S7).

**The site of Qiaozhen** (~2300–1800 BCE) in the city of Baoji in western Shaanxi is a Longshan site and covers an area of 150,000 m^2^ [9]. According to the publications, there are fragments consisting of cover tile, pan tile, and flat-pan tile. The cover tile is semi-cylindrical with differently shaped ends and is decorated with a basket pattern, while the pan tile and flat-pan tile are heavily fragmented. Archaeologists suspect that some tiles came from the deposits over the remaining limestone floor, which was probably the floor of the semi-subterranean house.

**The Jiantou site** (~2300–1800 BCE) is located in Jingchuan County, in southeastern Gansu [8]. A fragment of the cover tile is displayed. It is made of terracotta clay, and the convex surface is decorated with a basket pattern, indicating that it dates back to the Longshan period.

**The site of Jiangjiazui** (~2500–1800 BCE) is located in Lingtai County, in southeastern Gansu [10]. Archaeological investigation shows that it is a site extending from the Yangshao to the Longshan period, covering an area of 200,000 m^2^. Archaeologists have published papers about two cover tile fragments. They are decorated with basket patterns; specifically, two strips are respectively attached to two boundaries of the convex surface.

**The Lushanmao** **site** (~2350–2130 BCE) in the city of Yan’an, in northern Shaanxi, covers an area of about 200 ha [5]. According to the publications, at least 130 clay tile fragments, including cover and flat-pan tiles, were discovered on the ground and in the collapsed remains. The convex surface of the cover tiles is almost undecorated, except that both edges and the larger end have stripes, which are also observed in the Jiangjiazui tiles. In addition, two stripes along the edges do not extend to the smaller ends; we suspect that they also serve to control the overlap length. There is also a distinction between smaller and larger ends in the flat-pan tiles, with a strip on the concave surface of the larger ends (Fig. S6).

**The site of Shimao** (~2200–1550 BCE) is located in Shenmu County, in northern Shaanxi [11]. The total area of the complex is estimated at 400 ha and consists of three enclosures: Huangchengtai, the inner enclosure, and the outer enclosure. A foundation of rammed earth was found on the top of Huangchengtai and 200 tile fragments were excavated from the deposits around the rammed-earth walls of Huangchengtai. The short report has published a fragment and explained that it is a part of a cover tile.

In addition, some archaeologists stated that clay tiles were also found at the Matengkong site in Xi’an (in Shaanxi) and at the Taosi site in Xiangfen County, Shanxi. According to the report, there are three suspected fragments of cover tiles found in the deposits of the late Yangshao period (~2900 BCE) at the Matengkong site [12]. However, we argue that these three fragments belonged to tiles for two reasons: 1) they are too large to be tiles, because the restored widths are respectively about 17.2, 18, and 13 cm; 2) they are too few and too different to meet the requirements for laying composite tiles. As for the site of Taosi, the archaeologists found some fragments of slabs in the shape of a parallelogram or trapezoid in a ditch HG8 and claimed that they were tiles [13]. However, these fragments have nearly flat undersides with no sidewalls and are unfavorable for drainage and tiling. Some scholars speculated that they were not tiles but other building materials [14].

**Technological diversification between sites**

Clay tiles were found sporadically but concentrated on the Loess Plateau of Shaanxi and Gansu in the prehistoric period, and these sites shared the tradition of composite tile roofing. However, the available materials also show the diversity of tiles in prehistoric China. Their design, such as the different types and shapes, varies from site to site. For example, the main tile types at Lushanmao are flat-pan tiles, in contrast to Qiaocun where pan tiles are more common. The cover tiles of Lushanmao also differ from those in Qiaocun. They are grayish in color and are attached with clay nails that are edged with strips of clay. These differences indicated various methods of manufacture and imbrication, especially some designs related to the overlapping elements. However, we do not know whether such diversities originate from regional or chronological differences.

**S6.** The reconstruction of the virtual building and weight-loading testing

Since most tiles are highly fragmented, 3D modeling can help with reconstruction, and a computer simulation will evaluate the feasibility of composite tile roofing. Here, we would like to add a brief description of the virtual building, which refers to building 5 of the Lushanmao site.

The most likely number of cover and pan tiles at Qiaocun is 20 rows by 50 columns (including 10 columns of flat-pan tiles on two gable walls) corresponding to a house size plant of 12 x 12.87 m, which is approximately the size of building 5 at Lushanmao. Based on the above, the virtual building is built of rammed-earth walls with a bottom width of 1.8 m and a top width of 1.2 m; in addition, the height is roughly predicted to be about 5.4 m in consideration that the wall’s height is generally 2–3 times the thickness. The cross-section of the wall is isosceles-trapezoidal, and such a structure could achieve a better load-bearing effect. Since the front part of building 5 is slightly destroyed, there is no evidence of doorways. Therefore, we designed a door with a width of 2 m and a height of 3 m, which was placed on the central axis of a non-load-bearing wall to indicate the presence of the door. The roof is designed in the style of a flush gable roof, and its timber framework consists of beams and rafters. We have selected nine logs with a diameter of 30 cm and a length of 12.24 m as beams, while there are two types of rafters with a diameter of 10 cm, including type 1, with a length of 2.6 m at the eaves, and type 2, with a length of 2.3 m.

According to the measurements of the well-preserved samples, the weight of an average-sized cover plate is about 0.7 kg, while the weight of an average-sized pan tile is estimated at 3 kg. Based on the most probable figure, we estimate the weight of the tiled roof to be not less than 7.4 t. In addition, we also consider the snow load. The maximum snowfall in Gansu in the last 50 years is used as a reference, since the life cycle of civil architecture is generally 50 years. The highest value is now interrogated as 0.7 kN/m^2^, and one side of the roof covers an area of 85.68 m^2^. The product of the maximum snowfall and the total roof area corresponds to the snow load, which is a weight of 11,995.2 kg. We estimate that the weight for the force analysis is about 12 t. The experiment shows that the maximum deformation is 0.002 mm, which is within the tolerable range for the whole structure. Moreover, the stresses on the beam and rafter are lower than the yield strength, indicating that the deformation can be recovered even without loading (Fig. S8). In summary, the structure is sufficient to support such a roof load.

**S7.** The Greco-Etruscan tradition of tile-roofing techniques in Western Eurasia

The earliest Mediterranean tiles in early Helladic Greece were simple slab forms that may have imitated stone slabs. They were placed on roofs like fish scales. These tiles were often used for the “corridor houses,” large (150–300 m^2^), two-story, mudbrick and stone-walled buildings built in larger Early Helladic II sites (the medium-sized site of 10–20 ha) such as Lerna [15-17]. More elaborate composite tile settings first appeared in Greece in Late Helladic architecture. Although the break with the early technique of single-tile roofs is clear, they continued to be used for some special buildings, such as temples [18, 19]. Inspiration for two novel shapes of composite tiles at this stage may have come from cylindrical and rectangular drains [20, 21], and all these tiles were handmade without molding [22].

More than four centuries later, composite tiles reappeared in Archaic Greece (~700 BCE) [23]. During this period, roofing systems were vastly improved, both technically and decoratively. This first took the form of a new roofing technique known as Proto-Corinthian tiles, which combined concave pan tiles and convex cover tiles into a single unit [24]. Thereafter, two roof systems, Corinthian and Laconian, were the predominant styles in the Greek world. Differentiated ridge and eave tiles were also used. The techniques of molding were widespread, and on roofs with different tiles are found different types of molded decorations. Subsequently, composite tiles spread rapidly to Central Italy and the Black Sea region around 650–600 BCE[25, 26], with the further geographical expansion of such roofs to sites in Britain, France, and other parts of Europe during the Roman Empire (3^rd^ century) [27, 28].

In summary, there are two global centers for the origin and diffusion of clay tiles in both eastern and western Eurasia. In contrast to the frequent shifts and reinventions in the eastern Mediterranean region, where tiles were used on large buildings with mudbrick and stone walls in medium-sized sites of about 10–20 ha (Greco-Etruscan tradition), roof tiling techniques in East Asia demonstrate conspicuous continuity, with the Chinese Loess Plateau at the center (Longshan–Western Zhou tradition), where tiles were roofed on rammed-earth walls in the large central sites (more than 100 ha) (Table S5).

**S8**. Timber frame buildings and the *dougong* method

The timber framework is the main structure in ancient Chinese architecture, and there are two basic structural types including the *tailiang* (lifting beams) and *chuandou* (pillars and beams) [29, 30]. The *tailiang* is used more widely than the *chuandou*, and the structure of *tailiang* has been initially mature since the Spring and Autumn Period (771–476 BCE) [29].

Due to the hierarchy of buildings, the lifting-beams timber frame has different materials and combination methods. The best known is the *dougong* method, which only can be used in high-grade buildings. According to archaeological discoveries and studies, the *dougong* method has appeared since at least the early Western Zhou (1046–771 BCE), but it was widely used in important buildings until the Western Han Dynasty (202 BCE–8 CE). During the Tang Dynasty (618–907 CE), the *dougong* method tended to be unified [29].

**S9.** A bootstrap method to identify the larger and smaller ends of pan tiles

We use bootstrapping to identify the larger and smaller ends from a pan tile width and thickness dataset. The pan scales with ends were combined into a single dataset from which random values were selected. The larger value was then assigned to the dataset with the larger end, while the smaller value was assigned to the dataset with the smaller end. The two samples were then returned to the dataset, which was then resampled. After taking 1000 samples, the distributions of the larger and the smaller ends were estimated using the two separate datasets. As a control, the Bootstrap method was also applied to the dataset with the cover plates. The results are perfect for the dataset, which was classified by hand (Table S7).


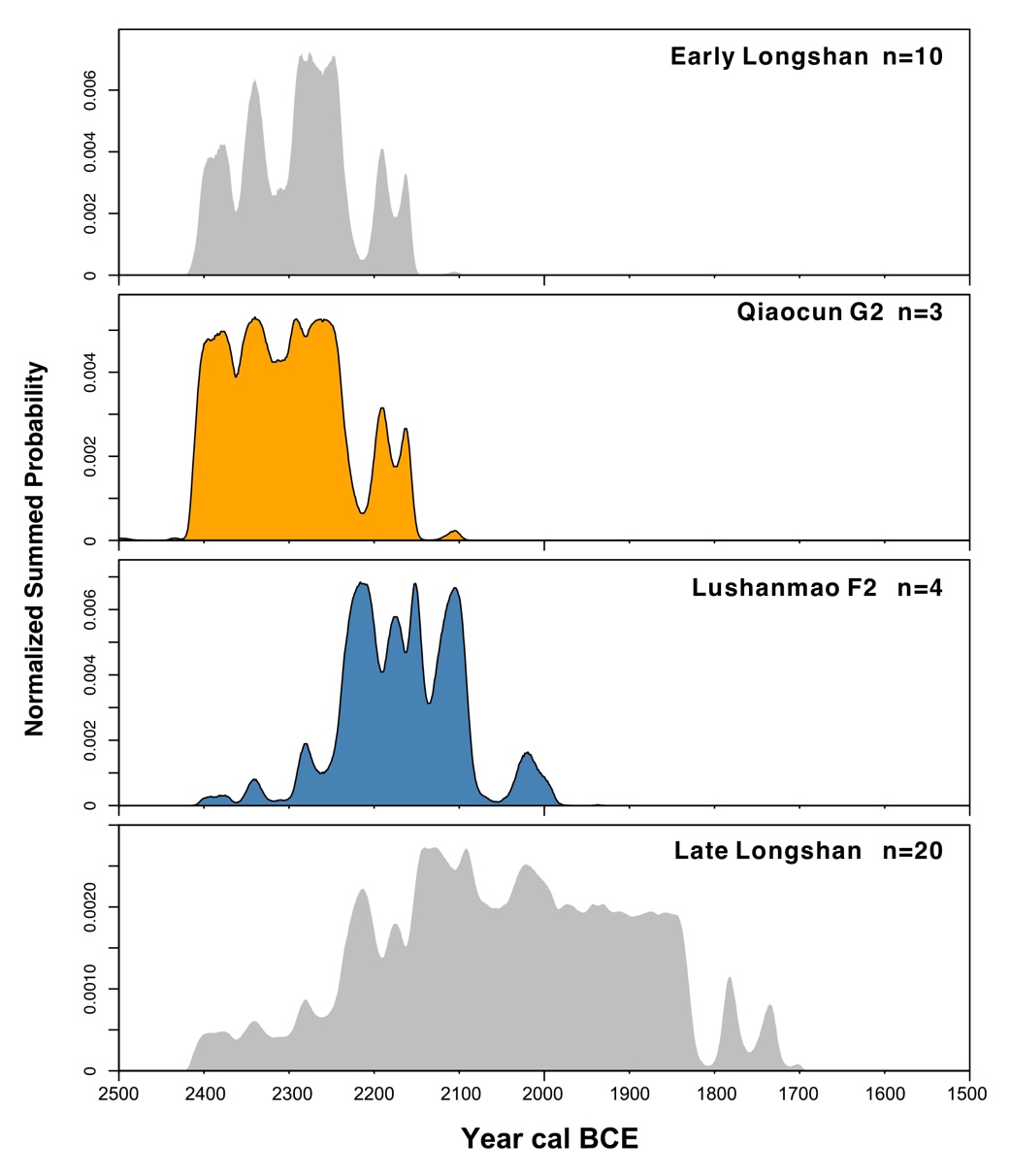


**Figure S1.** Normalized summed probabilities of radiocarbon dates of the Longshan sites on the Loess Plateau


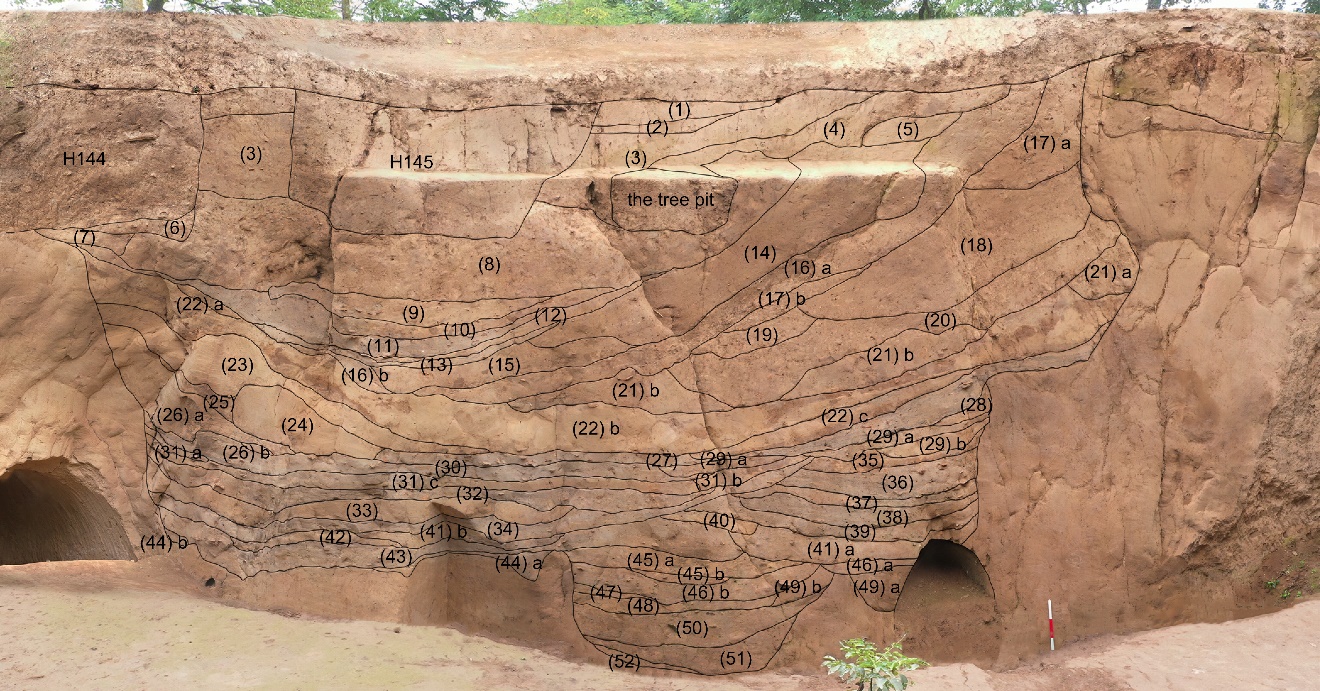


Figure S2. Profile of ditch G2 from Qiaocun site, where the tiles were excavated.





Figure S3. The *Yingzao Fashi*.


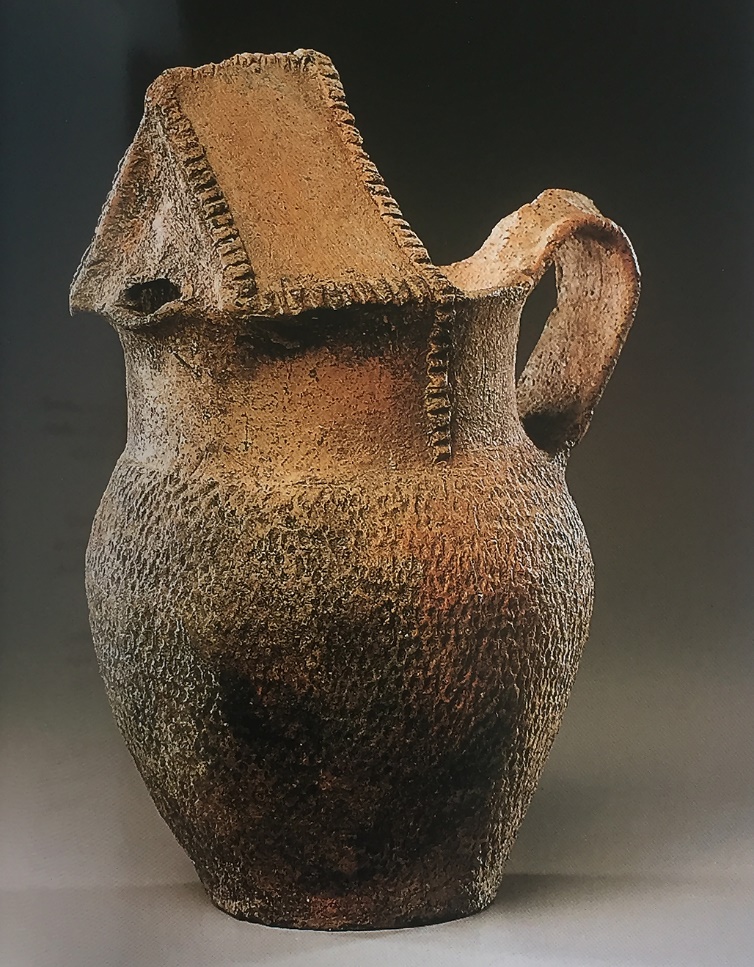


Figure S4. Jug with roof-shaped top.


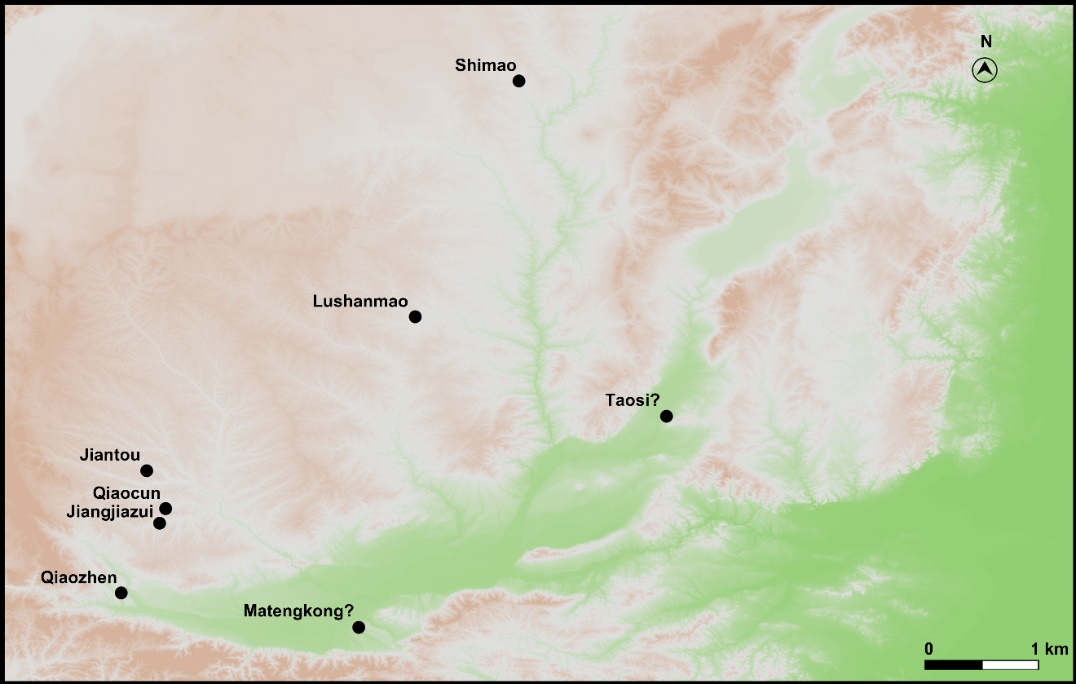


Figure S5. Map of sites with clay tiles in prehistoric China.


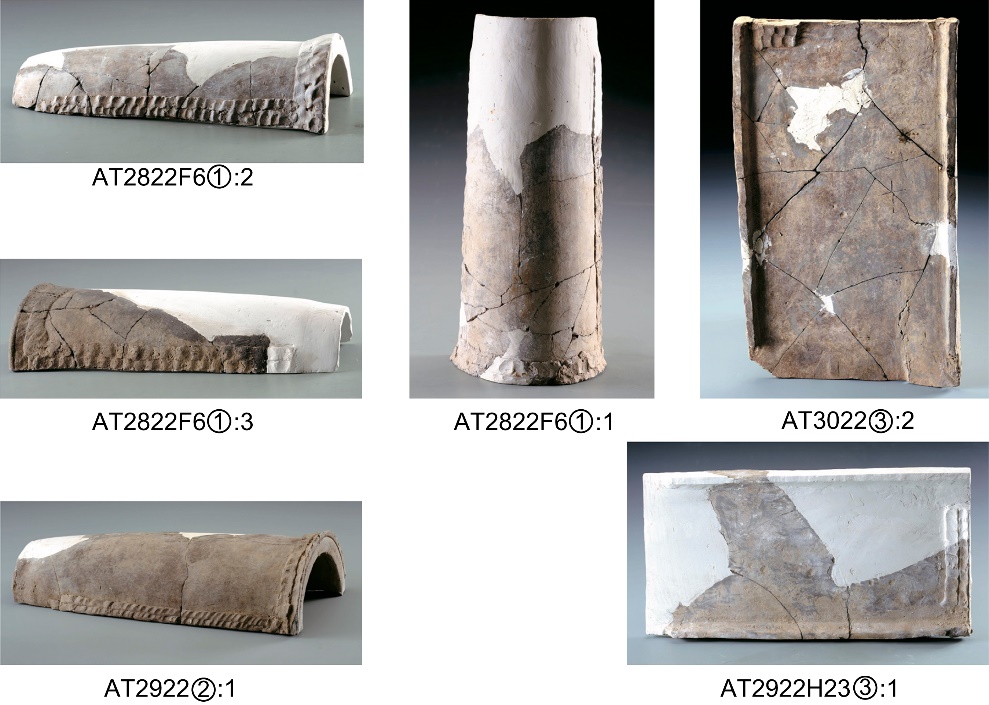


**Figure S6.** Composite tiles excavated from Lushanmao site.


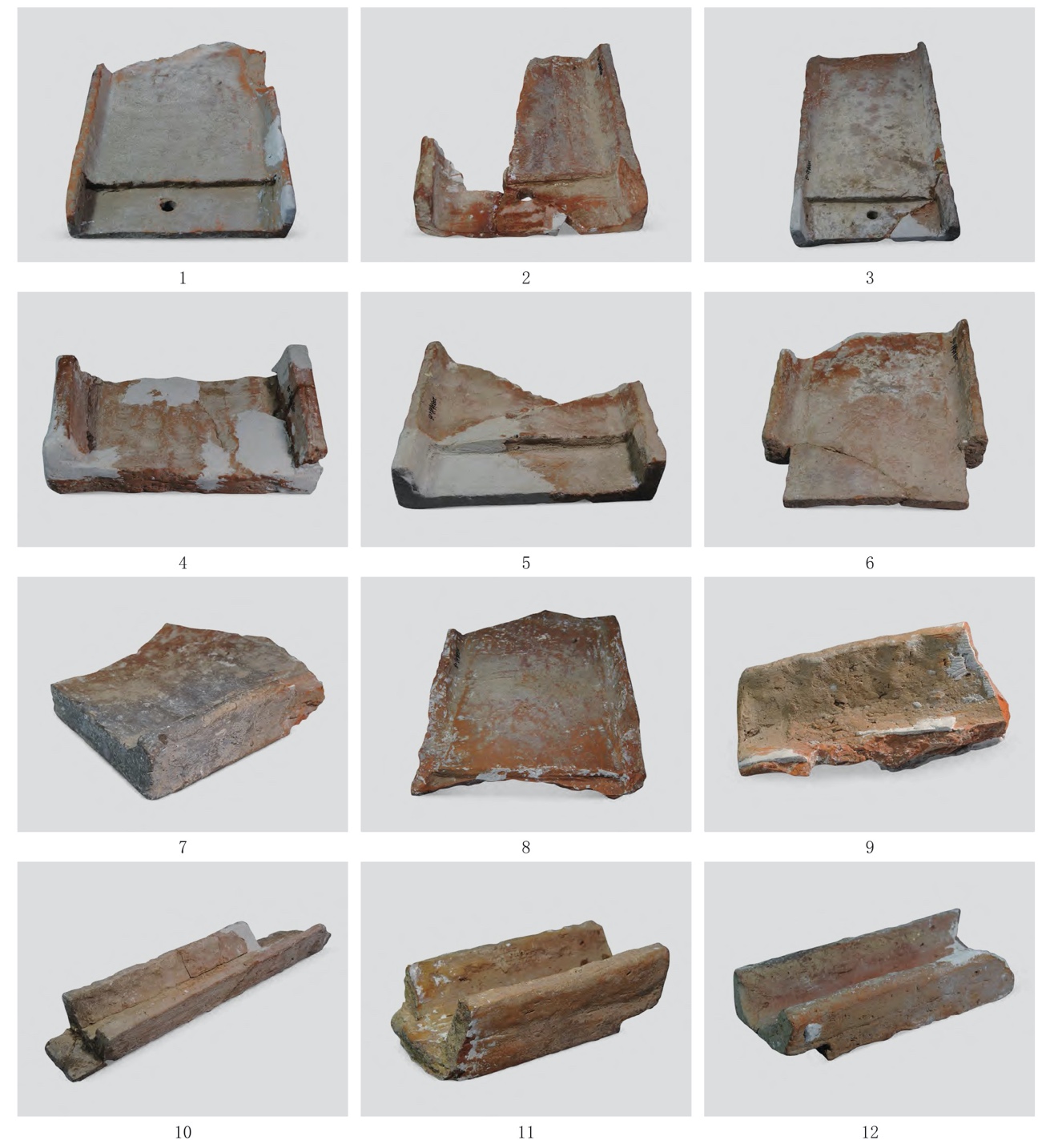


**Figure S7.** Flat-pan tiles collected from Qiaocun site suvery by Song et al. [8].


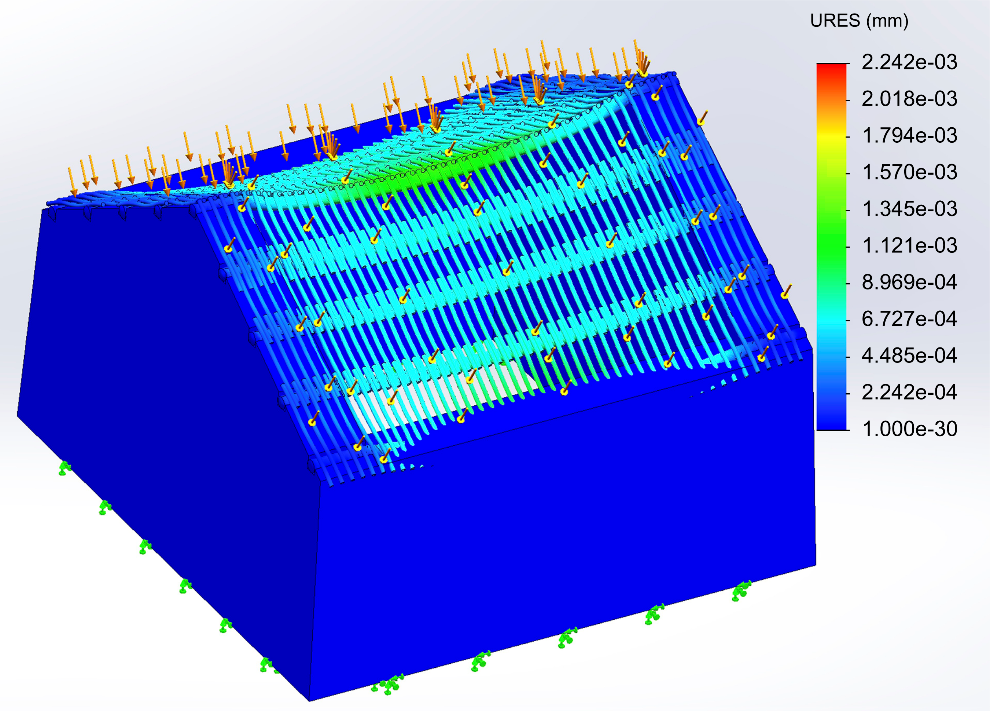


Figure S8. Stress diagram of the load-bearing test of tiled roof in simulated Qiaocun house.

**
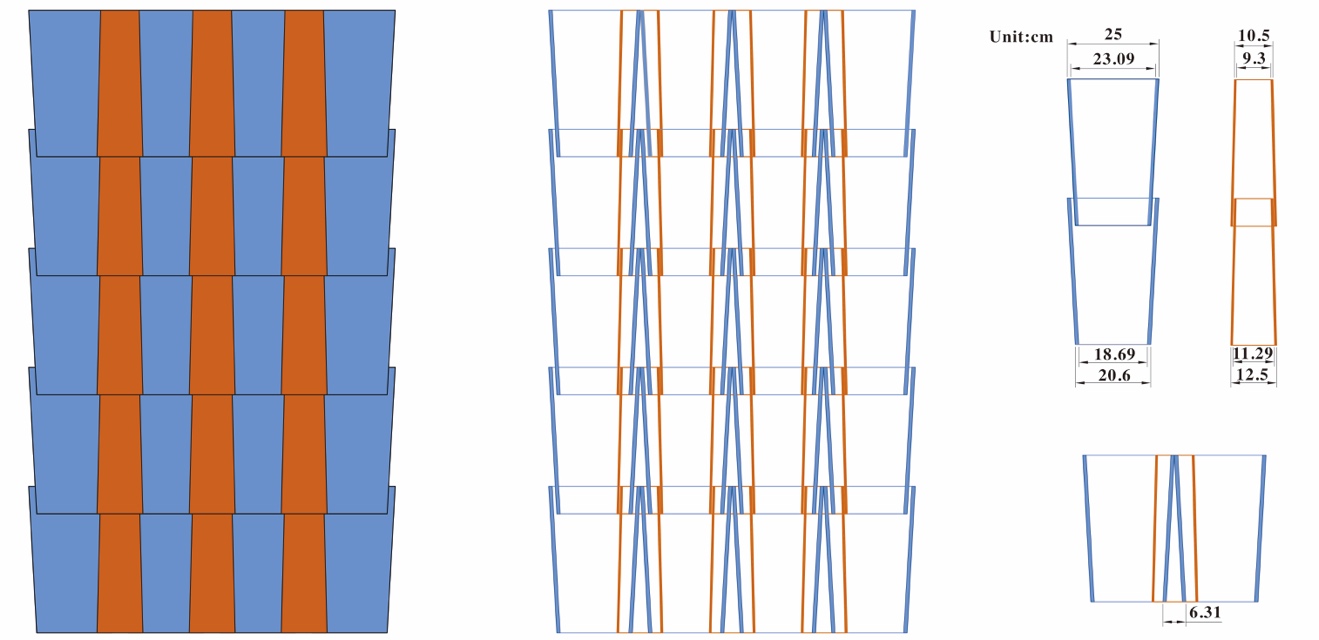
**

**Figure S9.** The method of installation of composite tiles in Qiaocun.

**Table S1.** Radiocarbon dates of the Longshan sites on the Loess Plateau

| Site | Deposit | Culture | Type | Radiocarbon age (BP) | | Calibrated dates (cal BCE) | |
| --- | --- | --- | --- | --- | --- | --- | --- |
|  |  |  |  | date | error | 1σ (68%) | 2σ (95%) |
| Qiaocun_early | G2_L10 | early Longshan | tooth | 3870 | 30 | 2353–2292 (35.0%) | 2462–2282 (87.8%) |
|  |  |  |  |  |  | 2407–2375 (17.3%) | 2251–2231 (5.1%) |
|  |  |  |  |  |  | 2452–2420 (16.0%) | 2222–2209 (2.6%) |
| Qiaocun_early | G2_L30 | early Longshan | bone | 3900 | 30 | 2461–2397 (41.5%) | 2468–2294 (95.4%) |
|  |  |  |  |  |  | 2386–2345 (26.8%) |  |
| Qiaocun_early | G2_L40 | early Longshan | tooth | 3850 | 30 | 2348–2280 (38.9%) | 2456–2270 (72.9%) |
|  |  |  |  |  |  | 2253–2209 (19.9%) | 2260–2204 (22.6%) |
|  |  |  |  |  |  | 2402–2381 (9.4%) |  |
| Qiaocun_late | 2018_M1_1 | late Longshan | tooth | 3710 | 30 | 2099–2037 (45.3%) | 2201–2025 (94.2%) |
|  |  |  |  |  |  | 2142–2115 (17.7%) | 1992–1984 (1.2%) |
|  |  |  |  |  |  | 2191–2182 (5.3%) |  |
| Qiaocun_late | 2018_M1_2 | late Longshan | bone | 3560 | 30 | 1952–1879 (61.9%) | 1980–1872 (71.2%) |
|  |  |  |  |  |  | 1839–1827 (6.4%) | 1848–1812 (11.5%) |
|  |  |  |  |  |  |  | 1806–1774 (6.0%) |
|  |  |  |  |  |  |  | 2020–1996 (4.7%) |
| Qiaocun_late | T4055K1_4 | late Longshan | millet | 3830 | 30 | 2306–2204 (58.0%) | 2355–2197 (80.8%) |
|  |  |  |  |  |  | 2341–2319 (10.2%) | 2408–2374 (7.2%) |
|  |  |  |  |  |  |  | 2453–2419 (4.6%) |
|  |  |  |  |  |  |  | 2169–2148 (3.1%) |
| Qiaocun_late | P258H1 | late Longshan | millet | 3900 | 30 | 2461–2397 (41.5%) | 2468–2294 (95.4%) |
|  |  |  |  |  |  | 2386–2345 (26.8%) |  |
| Qiaocun_late | P258H1 | late Longshan | rice | 3760 | 30 | 2206–2136 (54.9%) | 2238–2127 (65.2%) |
|  |  |  |  |  |  | 2276–2256 (9.8%) | 2287–2246 (15.4%) |
|  |  |  |  |  |  | 2076–2067 (3.6%) | 2092–2041 (14.9%) |
| Qiaocun_late | P173H1 | late Longshan | millet | 3580 | 30 | 1971–1888 (66.6%) | 2028–1878 (90.9%) |
|  |  |  |  |  |  | 2008–2005 (1.7%) | 1841–1824 (3.4%) |
|  |  |  |  |  |  |  | 1791–1782 (1.1%) |
| Qiaocun_late | P65H1 | late Longshan | millet | 3640 | 30 | 2036–1950 (63.7%) | 2059–1921 (76.2%) |
|  |  |  |  |  |  | 2112–2103 (4.6%) | 2135–2082 (18.0%) |
|  |  |  |  |  |  |  | 1912–1900 (1.3%) |
| Qiaocun_late | P_230 | late Longshan | wood | 3790 | 60 | 2304–2136 (62.0%) | 2411–2113 (80.6%) |
|  |  |  |  |  |  | 2339–2322 (4.0%) | 2101–2036 (10.5%) |
|  |  |  |  |  |  | 2077–2066 (2.3%) | 2456–2416 (4.4%) |
| Qiaocun_late | 78LXQH4 | late Longshan | wood | 3680 | 60 | 2142–2011 (55.2%) | 2206–1894 (93.8%) |
|  |  |  |  |  |  | 2001–1973 (10.1%) | 2276–2256 (1.6%) |
|  |  |  |  |  |  | 2191–2181 (3.0%) |  |
| Qiaocun_late | P_165 | late Longshan | wood | 3720 | 60 | 2201–2031 (68.4%) | 2296–1942 (95.4%) |
| Qiaocun_late | P_310 | late Longshan | wood | 3705 | 50 | 2146–2029 (58.7%) | 2209–1947 (92.2%) |
|  |  |  |  |  |  | 2196–2173 (9.6%) | 2281–2252 (3.1%) |
|  |  |  |  |  |  |  | 2227–2225 (0.2%) |
| Qiaocun_late | P_270 | late Longshan | wood | 3655 | 50 | 2051–1951 (46.7%) | 2146–1893 (92.3%) |
|  |  |  |  |  |  | 2131–2086 (21.5%) | 2196–2173 (3.1%) |
| Shuangan | H200⑥ | late Longshan | bone | 3640 | 30 | 2036–1950 (63.7%) | 2059–1921 (76.2%) |
|  |  |  |  |  |  | 2112–2103 (4.6%) | 2135–2082 (18.0%) |
|  |  |  |  |  |  |  | 1912–1900 (1.3%) |
| Shuangan | H283① | late Longshan | bone | 3630 | 30 | 2032–1946 (68.3%) | 2045–1896 (84.5%) |
|  |  |  |  |  |  |  | 2130–2089 (10.9%) |
| Shuangan | H349① | late Longshan | tooth | 3720 | 30 | 2096–2039 (39.4%) | 2204–2027 (95.4%) |
|  |  |  |  |  |  | 2146–2121 (15.3%) |  |
|  |  |  |  |  |  | 2196–2174 (13.6%) |  |
| Shimao | east gate | late Longshan | wood | 3730 | 25 | 2088–2047 (32.4%) | 2203–2110 (53.4%) |
|  |  |  |  |  |  | 2197–2171 (22.7%) | 2105–2035 (42.1%) |
|  |  |  |  |  |  | 2147–2131 (13.2%) |  |
| Shimao | east gate late layer | late Longshan | plaster | 3545 | 30 | 1936–1877 (47.8%) | 1973–1866 (58.2%) |
|  |  |  |  |  |  | 1842–1824 (12.9%) | 1853–1769 (36.3%) |
|  |  |  |  |  |  | 1793–1781 (7.6%) | 2010–2002 (1.0%) |
| Miaoliang | H49_D26 | early Longshan | bone | 3870 | 15 | 2350–2294 (41.2%) | 2458–2288 (95.4%) |
|  |  |  |  |  |  | 2404–2378 (17.8%) |  |
|  |  |  |  |  |  | 2441–2424 (9.3%) |  |
| Miaoliang | F20_D22 | early Longshan | bone | 3865 | 15 | 2349–2291 (48.0%) | 2457–2285 (93.2%) |
|  |  |  |  |  |  | 2404–2379 (16.7%) | 2248–2237 (2.3%) |
|  |  |  |  |  |  | 2434–2426 (3.6%) |  |
| Miaoliang | H49_D31 | early Longshan | bone | 3850 | 15 | 2344–2284 (54.5%) | 2353–2276 (57.1%) |
|  |  |  |  |  |  | 2248–2235 (8.7%) | 2256–2206 (21.3%) |
|  |  |  |  |  |  | 2397–2388 (5.1%) | 2406–2376 (11.6%) |
|  |  |  |  |  |  |  | 2452–2421 (5.4%) |
| Miaoliang | F19_D34 | early Longshan | bone | 3830 | 15 | 2256–2206 (49.4%) | 2344–2202 (95.1%) |
|  |  |  |  |  |  | 2297–2276 (18.8%) | 2393–2390 (0.4%) |
| Hongliang | IF4③_D2 | early Longshan | bone | 3865 | 15 | 2349–2291 (48.0%) | 2457–2285 (93.2%) |
|  |  |  |  |  |  | 2404–2379 (16.7%) | 2248–2237 (2.3%) |
|  |  |  |  |  |  | 2434–2426 (3.6%) |  |
| Hongliang | IIH26②_D23 | early Longshan | bone | 3860 | 15 | 2349–2287 (53.0%) | 2456–2282 (88.0%) |
|  |  |  |  |  |  | 2404–2379 (15.2%) | 2251–2232 (5.2%) |
|  |  |  |  |  |  |  | 2221–2209 (2.2%) |
| Hongliang | IF4③_D4 | early Longshan | bone | 3870 | 15 | 2350–2294 (41.2%) | 2458–2288 (95.4%) |
|  |  |  |  |  |  | 2404–2378 (17.8%) |  |
|  |  |  |  |  |  | 2441–2424 (9.3%) |  |
| Muzhuzhuliang | T1201M7 | late Longshan | bone | 3550 | 30 | 1943–1878 (52.6%) | 1976–1868 (63.1%) |
|  |  |  |  |  |  | 1841–1824 (10.7%) | 1851–1771 (30.3%) |
|  |  |  |  |  |  | 1792–1782 (4.9%) | 2014–1999 (2.0%) |
| Lushanmao | F2_1 | late Longshan | tooth | 3820 | 30 | 2299–2202 (64.2%) | 2350–2193 (82.5%) |
|  |  |  |  |  |  | 2337–2327 (4.1%) | 2177–2144 (6.8%) |
|  |  |  |  |  |  |  | 2405–2378 (4.2%) |
|  |  |  |  |  |  |  | 2447–2424 (1.9%) |
| Lushanmao | F2_2 | late Longshan | tooth | 3800 | 30 | 2287–2200 (61.5%) | 2343–2138 (95.4%) |
|  |  |  |  |  |  | 2163–2152 (6.7%) |  |
| Lushanmao | T2_3 | late Longshan | tooth | 3780 | 30 | 2180–2143 (28.3%) | 2297–2132 (91.2%) |
|  |  |  |  |  |  | 2282–2251 (22.0%) | 2085–2051 (4.2%) |
|  |  |  |  |  |  | 2210–2192 (12.4%) |  |
|  |  |  |  |  |  | 2231–2221 (5.6%) |  |
| Lushanmao | T2_4 | late Longshan | tooth | 3760 | 30 | 2206–2136 (54.9%) | 2238–2127 (65.2%) |
|  |  |  |  |  |  | 2276–2256 (9.8%) | 2287–2246 (15.4%) |
|  |  |  |  |  |  | 2076–2067 (3.6%) | 2092–2041 (14.9%) |

**Table S2A.** The statistic classification of pan tile specimens with one end reserved from the Qiaocun site.

|  | End with incisions | Smaller end | Larger end | Beyond recognition |
| --- | --- | --- | --- | --- |
| Number | 43 | 77 | 69 | 32 |
| Percentage | 19.46% | 34.84% | 31.22% | 14.47% |

**Table S2B**. The classification statistic of pan tiles with larger ends reserved from the Hetaoyuan site

|  | Pan tile decorated with two wavy lines | Pan tile decorated with one wavy line |
| --- | --- | --- |
| Pan tile (small) | 389 | 771 |
| Percentage | 33.53% | 66.47% |
| Pan tile (big) | 201 | 2233 |
| Percentage | 8.26% | 91.74% |

Note: Pan tiles decorated with two wavy lines are supposed to be placed in the first row along the eaves.

**Table S3.** Comparison between summary dimensions in Qiaocun and Hetaoyuan.

|  | N | Width (cm) | | N | Height (cm) | | N | Thickness (cm) | |
| --- | --- | --- | --- | --- | --- | --- | --- | --- | --- |
|  |  | M | CV^*^ |  | M | CV^*^ |  | M | CV^*^ |
| Cover (Q) | 215 | 11.50 | 14.69% | 215 | 5.60 | 19.28% | 215 | 0.86 | 18.66% |
| Cover (H) | 286 | 11.88 | 2% |  |  |  | 2246 | 1.65 | 9.57% |
| Pan (Q) | 39 | 21.93 | 15.69% | 182 | 4.90 | 21.55% | 573 | 1.03 | 20.22% |
| Pan (H) | 92 | 21.45 | 5.42% |  |  |  | 3586 | 1.63 | 10.70% |

Note: Q for Qiaocun, H for Hetaoyuan.

**Table S4.** Sites with early tiled roof houses in the world (2600 BCE–1000 CE).

| Site_ID | Site Name | 2600–2500 BCE | 2500–2000 BCE | 2000–1500 BCE | 1500–1000 BCE | 1000–500 BCE | 500–1 BCE | 1–500 CE | 500–1000 CE |
| --- | --- | --- | --- | --- | --- | --- | --- | --- | --- |
| 1 | Qiaocun |  | √ |  |  |  |  |  |  |
| 2 | Lushanmao |  | √ |  |  |  |  |  |  |
| 3 | Shimao |  | √ | √ |  |  |  |  |  |
| 4 | Zhouyuan |  |  |  |  | √ |  |  |  |
| 5 | Fenghao |  |  |  |  | √ |  |  |  |
| 6 | Hetaoyuan |  |  |  |  |  |  |  | √ |
| 7 | Yanxiadu |  |  |  |  |  | √ |  |  |
| 8 | Walled town of Kraskinskoe |  |  |  |  |  |  |  | √ |
| 9 | Pungnap Tosung |  |  |  |  |  |  | √ |  |
| 10 | Fujiwara Palace |  |  |  |  |  |  |  | √ |
| 11 | Asuka Temple |  |  |  |  |  |  |  | √ |
| 12 | Co Loa Citadel |  |  |  |  |  | √ |  |  |
| 13 | Buriram Province in Northeast Thailand |  |  |  |  |  |  |  | √ |
| 14 | Siem Reap Province in Cambodia |  |  |  |  |  |  |  | √ |
| 15 | Lerna | √ | √ |  |  |  |  |  |  |
| 16 | Thebes |  |  |  | √ |  |  |  |  |
| 17 | Gordion |  |  |  |  |  | √ |  |  |
| 18 | Meudon |  |  |  |  |  |  | √ |  |
| 19 | Cirencester |  |  |  |  |  |  | √ |  |

**Table S5.** Two tiled roof traditions in the eastern and western Eurasia.

| Eastern Eurasia | | | Western Eurasia | | |
| --- | --- | --- | --- | --- | --- |
| Date | Architectural structure and techniques | Roofing system | Date | Architectural structure and techniques | Roofing system |
| 2350  –  2130  BCE | Lushanmao site: rammed-earth walls with a bottom width of 1.8–2 m | cover and pan tiles, flat-pan tiles | 2650  –  2200  BCE | Lerna: unbaked mudbrick on a stone socle of five courses with a width of 0.8–0.9 m | tiles shaped like rectilinear slabs |
| 1800  –  1046  BCE | Erlitou site (1700–1530 BCE): columns ^(^^[[1]](#footnote-2))^ with a diameter of 0.2 m, wattle-and-daub walls, woods with a width of 0.19 m, and walls with a width of 0.75 m | no tiles | 1400  –  1200  BCE | Thebes: limestone socles with a width of 1.1–1.7 m, mudbrick elevations frequently with half-timbering | cover tiles, flat-pan tiles |
|  | Panlongcheng site (1500–1200 BCE): columns with a diameter of 0.2 m, wattle-and-daub walls, woods with a width of 0.2 m, and walls with a width of 0.75 m | no tiles |  |  |  |
|  | Sanxingdui site (1700–1200 BCE): no evidence for bearing structures | cover and pan tiles, flat-pan tiles |  |  |  |
| 1046  –  771  BCE | Zhouyuan site: columns ^(^^[[2]](#footnote-3))^ with a diameter range from 0.6 to 1.4 m, rammed-earth walls with a width of 0.8 m or brick walls | cover and pan tiles, flat-pan tiles, ridge tiles and half tile-ends | 1000  –  800  BCE | The Toumba Building at Lefkandi: stone foundations, but mudbrick, wood superstructure | no tiles |
| 771  –  221  BCE | Lingshou site (Zhongshan, 380–296 BC): columns with a diameter of 1.8 m and rammed-earth walls with a bottom width of 1.7 m | cover and pan tiles, half and tile-ends, and ridge tiles | 500  BCE | Temple of Aphaea at Aegina: stone foundations and supporting columns | Corinthian systems: flat-pan tiles, pitched cover tiles, ridge and eaves tiles |
|  | Yongcheng site (Qin, 677–383 BCE): rammed-earth walls, walls with a width of 1.2 m | cover and flat-pan tiles, half and tile-ends | 600  BCE | Temple of Hera at Olympia: stone foundations and supporting columns | Laconian systems: curved cover and pan tiles, ridge and eaves tiles |

**Table S6**. Possible restoration of the relationship between roof size, sherd numbers, and percentage of tiles.

| Row*Column | Tile number | | | Sherd percentage of tile | | |
| --- | --- | --- | --- | --- | --- | --- |
|  | Cover | Pan | Flat pan | Cover | Pan | Flat pan |
| 20*60 | 1214 | 1000 | 200 | 32.50% | 56.20% | 11.20% |
| 20*50 | 1008 | 800 | 200 | 32.40% | 54.10% | 13.50% |
| 20*40 | 803 | 600 | 200 | 32.30% | 50.70% | 16.90% |
| 20*30 | 597 | 400 | 200 | 32.10% | 45.20% | 22.60% |
| 15*60 | 919 | 750 | 150 | 32.70% | 56.10% | 11.20% |
| 15*50 | 763 | 600 | 150 | 32.60% | 53.90% | 13.50% |
| 15*40 | 608 | 450 | 150 | 32.50% | 50.60% | 16.90% |
| 15*30 | 452 | 300 | 150 | 32.40% | 45.10% | 22.50% |
| 10*60 | 624 | 500 | 100 | 33.10% | 55.70% | 11.10% |
| 10*50 | 518 | 400 | 100 | 33.00% | 53.60% | 13.40% |
| 10*40 | 413 | 300 | 100 | 33.00% | 50.30% | 16.80% |
| 10*30 | 307 | 200 | 100 | 32.80% | 44.80% | 22.40% |

Note: The breakage rate is 2.08.

**Table S7**. Statistics of cover tiles for the larger and smaller ends with a bootstrap method.

| Samples | Width of larger end | | Width of smaller end | |
| --- | --- | --- | --- | --- |
|  | Mean | Variance | Mean | Variance |
| Archeological sherd | 12.47 | 2.66 | 10.58 | 2.71 |
| Sample 1 | 12.63 | 2.73 | 10.38 | 2.11 |
| Sample 2 | 12.6 | 2.4 | 10.48 | 2.17 |
| Sample 3 | 12.55 | 2.8 | 10.26 | 2.52 |
| Sample 4 | 12.58 | 2.43 | 10.31 | 2.37 |
| Sample 5 | 12.53 | 2.5 | 10.31 | 2.14 |

Dataset S1 (separate file). Information on tile fragments with morphological characteristics.

Dataset S2 (separate file). Priority order of tile interlocking.

**References:**

1. Zhao, J., Zhou, J., Li, W. & Zhang, H. Preliminary report on the excavation of zone I at the Qiaocun site in Lingtai County, Gansu. *Kaogu yu wenwu* (*Archaeol. Cult. Relics)* **2**, 14–25 (2022).

2. Liu, H., Zhu, R., Song, G. & Qiao, L. Compound-sites of the Han period at Sanyangzhuang in Neihuang, Henan. *Kaogu (Archaeology)* **7**, 34–37 (2004).

3. Peng, M. *Study on architecture tilework in Northern Dynasties: The collation and research of tiles excavated from a Northern Qi stupa relic in Yecheng Site* (Institute of Archaeology, Chinese Academy of Social Sciences, 2017).

4. Rawson, J. China and the steppe: Reception and resistance. *Antiquity* **91**, 375–388. <http://doi.org/10.15184/aqy.2016.276> (2017).

5. Ma, M. *et al.* The Lushanmao site of the neolithic age in Yan’an City, Shaanxi. *Kaogu (Archaeology)* **7**, 29–45 (2019).

6. Li, W. *A study of the pottery-making technology in ancient China* (Science Press, 1996).

7. Wang, X. Research on the pottery-making technology in Southern Shanxi of the late neolithic period, *Zhongyuan wenwu (Cult. Relics Cent. Chin.)* **3**, 58–66 (2022).

8. Song, J., Chang, J. & Ma, M. Research on the ceramic tiles during the Longshan Era on the Loess Plateau. *Kaogu yu wenwu* (*Archaeol. Cult. Relics)* **2**, 119–131 (2022).

9. Liu, J. Building materials of the Longshan period in Baoji city. *Wenwu (Cult. Relics)* **3**, 44–45 (2011).

10. Song, J. A regional survey in the Daxi river valley. *Xibu kaogu (West. Archaeol.)* **1**, 29–70 (2017).

11. Sun, Z. *et al.* The Huangchengtai Locality of the Shimao Ancient City Site in Shenmu County, Shaanxi. *Kaogu (Archaeology)* **7**, 46–56 (2017).

12. Wang, Z. The late Yangshao cover tiles at the Matengkong site, Xi’an. <https://mp.weixin.qq.com/s/BJjQgh3FZuGmX8LZutc6Kw> (2022).

13. Yan, Z. & He, N. Excavation on the City-site at Taosi, Xiangfen, Shanxi in 2002. *Kaogu xuebao (Acta Archaeol. Sin.)* **3**, 307–346 (2005).

14. Chen, C. Doubt about ceramic plate in Tao Temple regarded as slate. *Hengyang shifan daxue xuebao (J. Hengyang Norm. Univ.)* **4**, 85–88 (2014).

15. Jazwa, K. A. The construction of early Helladic ii ceramic roofing tiles from Mitrou, Greece: Influence and interaction. *Mediterr. Archaeol. Archaeom.* **18**, 153–173; 10.5281/zenodo.1297155 (2018).

16. Winter, N. A. *Greek architectural terracottas: from the Prehistoric to the end of the Archaic Period* (Clarendon Press, 1993).

17. Shaw, J. W. Sequencing the EH II “Corridor Houses.” *Annu. Br. Sch. Athens* **102**, 137–151; 10.1017/S0068245400021456 (2007).

18. Burke, B., Burns, B. & Charami, A. Eastern Boeotia archaeological project: Preliminary report on excavations 2011–2013. *Teiresias* **43**, 9–25 (2013).

19. Burke, B. *et al.* Fieldwork at Ancient Eleon in Boeotia, 2011–2018. *Am. J. Archaeol.* **124**, 441–476; 10.3764/aja.124.3.0441 (2020).

20. Aravantinos, V. L., Fappas, I. & Galanakis, Y. Atop the Kadmeia: Mycenean roof tiles from Thebes in context. *Annu. Br. Sch. Athens* **115**, 175–245. 10.1017/S006824542000009X (2020).

21. Sapirstein, P. *The emergence of ceramic roof tiles in Archaic Greek architecture* (Cornell University, 2008).

22. Lakovidis, S. E. Mycenaean roofs: Form and construction in *L’Habitat Égéen Préhistorique* (eds Darcque, P. & Treuil, R.) 47–60 (École Française d’Athènes, 1990).

23. Winter, N. A. Defining regional styles in Archaic Greek architectural terracottas. *Hesperia* **59**, 13–32; 10.2307/148121 (1990).

24. Sapirstein, P. How the Corinthians manufactured their first roof tiles. *Hesperia* **78**, 195–229; 10.2972/hesp.78.2.195 (2009).

25. Glendinning, M. A mid-sixth-century tile roof system at Gordion. *Hesperia* **65**, 99–119; 10.2307/148462 (1996).

26. Munteanu, C., & Vochiţu, A. Roof tiles from the Ancient Greek shipwreck “Mangalia B”, Black Sea Coast, Romania. *Int. J. Naut. Archaeol.* **39**, 407–412; 10.1111/j.1095-9270.2010.00279.x (2010).

27. Mc Whirr, A., & Viner, D. The production and distribution of tiles in Roman Britain with particular reference to the Cirencester region. *Britannia* **9**, 359–377; 10.2307/525952 (1978).

28. Barat, Y. A tile-making workshop from the roman period (3rd century) at Meudon (Hauts-de-Seine). *Revue archéologique du Centre de la France* **41**, 225–237; 10.3406/racf.2002.2909 (2002).

29. Liu, D. *History of Ancient Chinese Architecture* (China Architecture & Building Press, 2003).

30. Bai, Y. & Sun, Y. Terminologies and the research on Chinese architectural history a historiographical investigation on "chuandou " and tailiang". *Jianzhu Xuebao (Archit. J.)* 12, 68–72 (2019).

1. The columns surrounded the house. [↑](#footnote-ref-2)
2. The columns were located in the interior to support the roof and divide the space. [↑](#footnote-ref-3)
